# Pharmacokinetic Evaluation of a Single Intramuscular High Dose versus an Oral Long-Term Supplementation of Cholecalciferol

**DOI:** 10.1371/journal.pone.0169620

**Published:** 2017-01-23

**Authors:** Katharina Wylon, Gennadiy Drozdenko, Alexander Krannich, Guido Heine, Sabine Dölle, Margitta Worm

**Affiliations:** 1 Klinik für Dermatologie, Venerologie und Allergologie, Charité Universitätsmedizin Berlin, Berlin, Germany; 2 Koordinierungszentrum für Klinische Studien, Charité Universitätsmedizin Berlin, Berlin, Germany; Cardiff University, UNITED KINGDOM

## Abstract

**Background and Objectives:**

Vitamin D deficiency is frequent during the winter and occurs throughout the year in the elderly or patients suffering from autoimmune diseases. The objective of this study was to evaluate the pharmacokinetic properties of oral supplementation versus a single intramuscular injection of cholecalciferol in healthy individuals.

**Research design and methods:**

Up to 8,000 I.U. oral cholecalciferol was administered daily for 84 days in a 4 week dose-escalation setting to vitamin D deficient individuals. In another cohort, a single intramuscular injection of 100,000 I.U. cholecalciferol was given. In both cohorts, individuals without vitamin D intake served as the comparison group. 25-hydroxyvitamin D (25(OH)D) concentrations were measured in all individuals at defined time points throughout the studies.

**Results:**

The mean 25(OH)D serum concentration increased significantly after oral cholecalciferol intake compared to the control group (day 28: 83.4 nmol/l and 42.5 nmol/l; day 56: 127.4 nmol/l and 37.3 nmol/l; day 84: 159.7 nmol/l and 30.0 nmol/l). In individuals receiving 100,000 I.U. cholecalciferol intramuscular, the mean 25(OH)D serum concentration peaked after 4 weeks measuring 70.9 nmol/l compared to 32.7 nmol/l in the placebo group (p = 0.002). The increase of 25(OH)D serum concentrations after 28 days was comparable between both routes of administration (p = 0.264).

**Conclusions:**

Oral and intramuscular cholecalciferol supplementation effectively increased serum 25(OH)D concentrations.

## Introduction

Vitamin D deficiency is a frequent medical condition, not only in the elderly but also in young adults, depending on lifestyle factors [1–3]. The major sources of vitamin D (chemical cholecalciferol) are the cutaneous synthesis upon sunlight exposure and, to a minor extent, dietary intake. The active form of vitamin D is a fat-soluble seco-steroid hormone [4]. When synthesized in the skin or provided through the diet, vitamin D is biologically inactive. It is hydroxylated twice into (a) the storage metabolite 25-hydroxyvitmamin D (25(OH)D) and (b) the biologically active metabolite 1.25-dihydroxyvitamin D (calcitriol) [5]. The 25(OH)D has a circulating half-life of 3 weeks [6] and is commonly used to determine the vitamin D status [7]. Calcitriol is known to regulate intestinal calcium absorption, serum calcium and phosphate homeostasis, bone mineralization and immune regulation [8, 9]. The optimal dosage, frequency and route of administration to reach sufficient vitamin D levels in the blood (25(OH)D > 50 nmol/l) are still controversially discussed [6]. Different administration routes are used to increase systemic vitamin D concentrations, e.g. oral and intramuscular (i.m.). The oral supplementation is the first-line vitamin D deficiency treatment. If 25(OH)D serum concentrations do not increase after adequate substitution, an i.m. injection with cholecalciferol is indicated. The possible reasons for oral vitamin D resistance include malabsorption, liver—or kidney failure and obesity [10]. Single, large vitamin D doses were studied without determining the optimal dosage, or route of administration. Until now no general recommendation is available.

This study had been designed to assess peak vitamin D concentrations after a 100,000 I.U. single dose application in comparison to monthly increasing oral cholecalciferol substitution.

## Patients and Methods

### Oral vitamin D supplementation

In the first cohort, 43 healthy subjects between the age of 18 and 60 years were enrolled in an open label study (Table 1) [11]. The exclusion criteria were a lack of consent, incompliance, scheduled sun tanning or UV exposure, a positive history of sarcoidosis, hypercalcemia, serum creatinine concentration > 1 mg/dl, nephrolithiasis, pregnancy and lactation, diseases of the cardiovascular system, cancer, malabsorption or chronic infection. Individuals of the vitamin D group (n = 25) received monthly increasing doses of 2,000 I.U. (50μg, week 1 to 4), 4,000 I.U. (100μg, week 5 to 8) and 8,000 I.U. (200μg, week 9 to 12) of cholecalciferol per day. Healthy individuals without vitamin D intake served as a control group (n = 18). Both study groups were comparable with respect to age, gender, and basal serum 25(OH)D concentrations (Table 1). Blood samples for serum analysis were drawn at baseline and after 4, 8 and 12 weeks. Serum 25(OH)D levels were measured using the serum 25(OH)D ELISA kit (IDS Hamburg, Germany).

**Table 1:** Baseline characteristics of the patients with oral and intramuscular vitamin D supplementation. Values given as mean and standard deviation; p-values calculated using Students-T-Test, n.s. = not significant, n.a. = not applicable.

Study	oral	oral	p-value	i.m.	i.m.	p-value
Characteristics	Vitamin D	Placebo	n.a.	Vitamin D	Placebo	n.a.
**Number (n)**	25	18	n.a.	12	6	n.a.
**Sex (f /m)**	9 / 16	7 / 11	n.a.	8 / 4	4 / 2	n.a.
**Age (years)**	33.4 ± 6.6	31.7 ± 5.2	n.s.	34.9 ± 9.1	36 ± 13	n.s.
**25(OH)D (nmol/l)**	40.0 ± 12.9	46.3 ± 14.0	n.s.	33.0 ± 8.5	42.9 ± 6.1	n.s.
**Body mass index (BMI)**	23.2 ± 3.3	23.0 ± 3.5	n.s.	22.2 ± 3.2	22.3 ± 3.6	n.s.

### Intramuscular vitamin D supplementation

**Fig 1:**
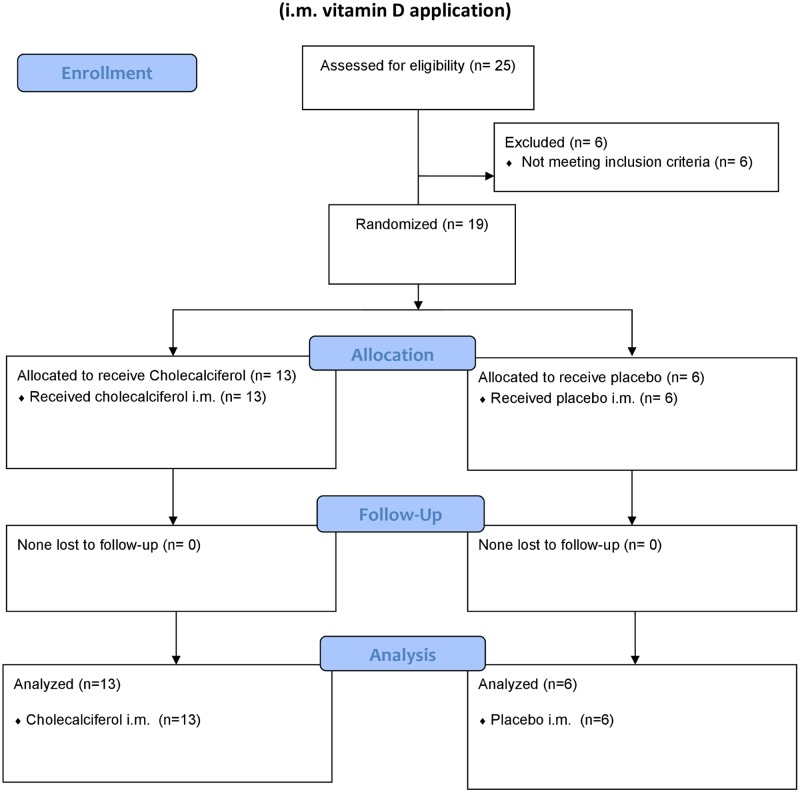
Consort flow chart. I.m. vitamin D application.

The second cohort of 18 healthy vitamin D deficient (<55 nmol/l as per protocol) subjects was randomized in a placebo-controlled pilot study (Table 1). Twelve women and 6 men between 18 and 60 years of age were recruited and enrolled if the inclusion and exclusion criteria as mentioned above were met. The cohorts randomization into vitamin D or placebo groups (ratio 2:1) performed by assigning the individuals in the order of appearance to a gender-specific list generated by an external pharmacist (Charité Berlin, Germany) (Fig 1).

The study medication was applied by an unblinded staff member to 12 individuals receiving an i.m. injection with 100,000 I.U. cholecalciferol (D3-Vicotrat^®^, Heyl Germany) and 6 subjects receiving a sodium chloride injection (Braun Melsungen, Germany, Placebo).

Both study groups were comparable according to age, ethnicity, weight and height (Table 1). Pharmacokinetics were monitored by 25(OH)D serum concentrations which were measured by our laboratory (Labor Berlin—Charité Vivantes GmbH).

Both clinical studies were approved by the ethical committee (Ethik-Kommission des Landes Berlin) and conducted according to the principles expressed in the Helsinki Declaration. A written and oral informed consent had been obtained from the participants before any investigation was performed. Both studies were performed during the winter months (October to March).

## Statistical Methods

Statistical evaluations were performed with GraphPad Prism 5 (GraphPad Software, Inc., CA, USA) and SPSS21 (IBM, NY, USA). Normal distribution was tested by the Kolmogorov-Smirnov test. The data for the serum 25(OH)D concentrations were analysed and values are shown as mean ± SD (standard deviation). 25(OH)D increase from the baseline visit to the 28th day between the oral vitamin D and the i.m. verum group was analysed using a two-sided t-test for independent groups. P-values (p) ≤ 0.05 were considered to be statistically significant. Effect size (dCohen) and confidence intervals (CI) were calculated as described elsewhere [12]. Serum 25(OH)D increase inter-group comparison was tested with Whitney-Mann-U-Test.

## Results

### Oral vitamin D intake efficiently increased serum 25(OH)D concentrations, but not in the control group

Vitamin D deficiency is defined as a serum 25(OH)D level of less than 50 nmol/l(10), based on the recommendations of the Institute of Medicine (IOM) [13]. Others and our group observed a seasonal variation in serum 25(OH)D concentrations in the latitude of Berlin, Germany (approximately 52°N) [14]. During the winter months, serum 25(OH)D levels are significantly lower due to an insufficient amount of UV-light (October-March: UV-index ≤ 2) and lacking skin exposure by low temperatures [15], which eliminates the bias of UV-mediated vitamin D synthesis.

As expected, the baseline 25(OH)D serum concentrations in the control group without vitamin D intake decreased during the study period (mean: day 0 = 46.3 nmol/l, day 28 = 42.5 nmol/l, day 56 = 37.3 nmol/l, day 84 = 30.0; p<0.001compared to the baseline (Table 2).

**Table 2:** Serum 25(OH)D concentrations during oral vitamin D supplementation. Data shown as mean ± SD, number of individuals, n; p-values calculated by Students-T-Test, effect size calculated by d_Cohen_ and 95% confidence intervals. Not applicable = n.a.

Day of the study (daily vit.D in I.U.)	Vitamin D group	Control group	p-value	Effect size d_Cohen_ (CI 95%)
0	40.0 ± 12.9, n = 25	46.3 ± 14.0, n = 17	0.23	n.a.
28 (2000 I.U)	83.4 ± 14.5, n = 25	42.5 ± 13.4, n = 18	< 0.001	2.93 (2.04–3.82)
56 (4000 I.U)	127.4 ± 38.1, n = 25	37.3 ± 14.6, n = 18	< 0.001	3.00 (2.10–3.90)
84 (8000 I.U)	159.7 ± 28.7, n = 10	30.0 ± 11.5, n = 18	< 0.001	6.59 (4.63–8.56)
84 (vit.D stopped at day 56)	96.1 ± 20.1, n = 15	30.0 ± 11.5, n = 18	< 0.001	4.19 (2.95–5.43)

In the vitamin D group, the 25(OH)D serum concentrations increased from a mean baseline level of 40.0 nmol/l during 4-week intervals with the oral intake of 2,000 I.U. cholecalciferol per day (total dose of 56,000 I.U.) to 83.4 nmol/l (p<0.001, dCohen compared to control = 2.93), after an additional 4 week with 4.000 I.U. cholecalciferol per day (total 112,000 I.U.) to 127.4 nmol/l (p<0.001, dCohen = 3.00), and finally after the last 4 week with 8,000 I.U. per day (total 224,000 I.U.) to 159.7 nmol/l (p<0.001, dCohen = 6.59, n = 10). In the subgroup reaching up to 110.0 nmol/l serum 25(OH)D at day 56, vitamin D intake was stopped to prevent potentially toxic levels and decreased values were determined on day 84 (96.1 nmol/l (p<0.001, dCohen = 4.19, n = 15).

Intramuscular vitamin D supplementation significantly increased serum 25(OH)D concentrations for 3 months, in contrast to placebo.

25(OH)D serum concentration was measured before the single cholecalciferol injection of 100,000 I.U. on day 3, day 7, day 14, day 21, day 28, day 42, day 56 and day 84 after the administration (Table 3).

**Table 3:** Serum 25(OH)D concentrations during intramuscular vitamin D supplementation. Data shown as mean ± SD, number of individuals, n; p-values calculated by Students-T-Test, effect size calculated by d_Cohen_ and 95% confidence intervals. Not applicable = n.a.

Days after vit D injection	Vitamin D (i.m.) n = 12	Placebo (i.m.) n = 6	p-value	Effect size d_Cohen_ (CI 95%)
0	32.2 ± 8.9	42.9 ± 6.7	0.03	n.a.
7	57.8 ± 13.8	39.8 ± 8.4	0.01	1.50 (0.41–2.60)
28	70.9 ± 21.6	37.0 ± 7.1	0.002	1.92 (0.76–3.09)
56	58.0 ± 16.7	32.7 ± 8.7	0.004	1.77 (0.63–2.91)
84	75.2 ± 19.9	49.7 ± 9.1	0.009	1.55 (0.44–2.65)

The baseline 25(OH)D serum concentrations were comparable in the vitamin D group measuring 32.2 nmol/l ± 8.9 nmol/l (range: <12.5 nmol/l to 49.7 nmol/l) and in the placebo group measuring 42.9 nmol/l ± 6.7 nmol/l (range: 38.3 nmol/l to 55.1 nmol/l). 3 days after vitamin D administration, the serum concentration increased by 16.8 nmol/l in the vitamin D group. After 7 days, 25(OH)D serum concentrations increased to a mean of 57.8 nmol/l ± 13.8 nmol/l in the vitamin D group, whereas it decreased to a mean of 39.8 nmol/l in the placebo treated group (Table 3). On day 28, the 25(OH)D serum concentrations increased significantly and peaked in the vitamin D treated group with a mean of 70.9 nmol/l and remained low in the placebo treated individuals (mean 37.0 nmol/l, p = 0.002). After 2 months, 25(OH)D serum concentrations was again decreased to 58.0 nmol/l in the vitamin D group and 32.7 nmol/l in the placebo treated group. Finally, 3 months after the injection, with the beginning of the UV rich season 25(OH)D (April, UV-index 4–5), serum concentrations increased in the vitamin D (75.2 nmol/l) as well as in the placebo group (49.7 nmol/l).

### Influencing factors and tolerability

There were no correlations of the 25(OH)D serum levels between gender, age or body mass index (BMI). No side effects or adverse events related to the investigational drug were reported throughout both clinical studies. The serum calcium and phosphate levels were monitored for safety purposes and remained stable throughout the study (data not shown). 5 individuals showed a mild hypophosphatemia which was not related to vitamin D supplementation.

A comparable increase in serum 25(OH)D concentration upon 4 weeks of oral and intramuscular supplementation.

**Fig 2:**
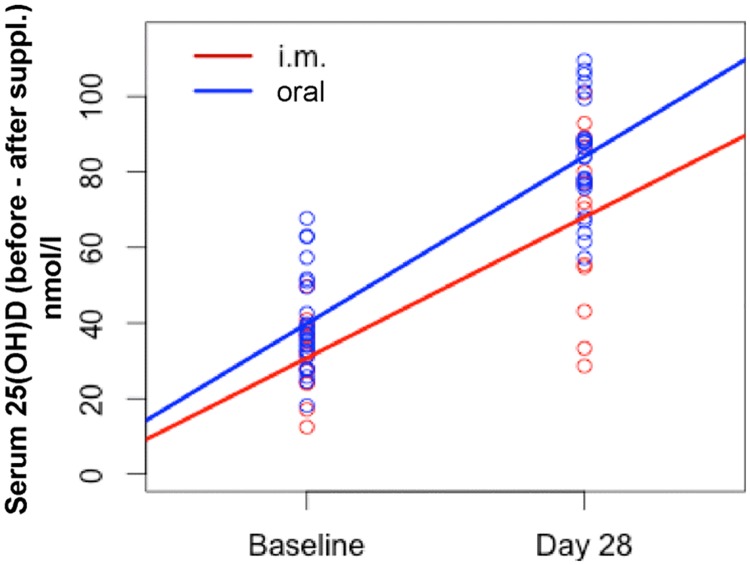
Serum 25(OH)D concentration at the baseline and day 28. Serum 25(OH)D was determined at the baseline and day 28 upon oral and i.m. supplementation.

25(OH)D serum concentrations increased significantly upon both, the oral and i.m. route of administration (Fig 2).

**Fig 3:**
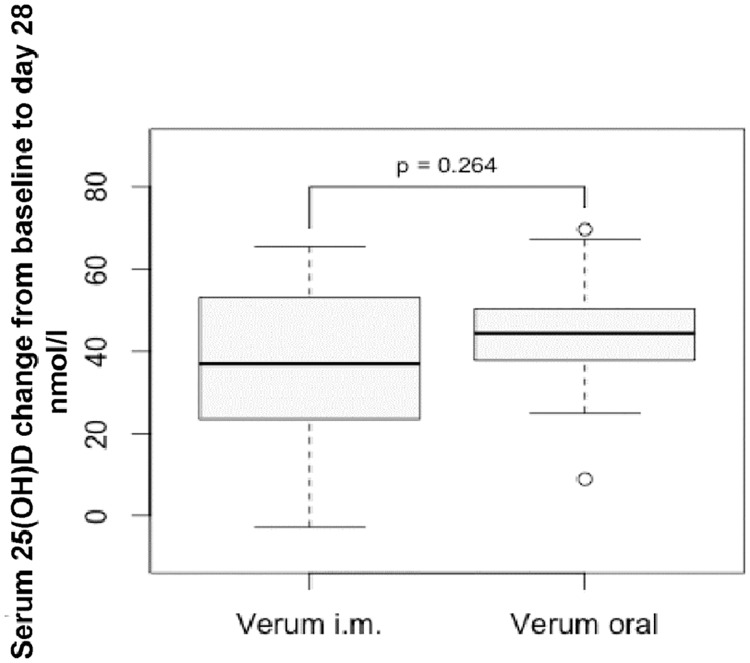
Serum 25(OH)D concentration after oral and i.m. vitamin D supplementation. Comparable increase of serum 25(OH)D concentration at the baseline and day 28 in the oral and i.m. supplementation group. Data shown as median, interquartile ranges and overall range with p-value calculated by Whitney-Mann-U-Test.

Next, we compared the 25(OH)D serum concentration increase from the baseline to the day 28 between oral and i.m. supplementation (Fig 3).

The mean 25(OH)D increase in the oral group measured 44.2 nmol/l ± 13.1 nmol/l and the i.m. group was 37.2 nmol/l ± 19.6 nmol/l. Statistical analysis revealed no significant difference in 25(OH)D increase between oral and i.m. supplementation groups (Fig 3, p = 0.264).

## Discussion

Vitamin D deficiency is prevalent in the population of higher latitudes [10, 16, 17]. Several treatment approaches using oral and injectable vitamin D have been proposed in the literature for vitamin D deficiency correction. It has been shown that daily oral [18–20] or a single high dose [21, 22] supplementation effectively restores the vitamin D status. Upon certain circumstances, e.g. limited intestinal resorption, i.m. injections are the preferred route of administration [23, 24]. Our data shows that one month after an i.m. injection of 100,000 I.U. cholecalciferol the 25(OH)D serum concentrations increased to a mean of 70.9 nmol/l. As approximately 50% of the individuals still remained vitamin D deficient after a single dose, the data suggest that a higher dose is most likely more effective and probably safe [1, 23, 25]. Only by future prospective randomized clinical trials with different doses given, e.g. 100,000 I.U., 150,000 I.U., 200,000 I.U. or even higher will clarify the optimal dose of i.m. vitamin D supplementation. Otherwise, analysis of repeated administration of 100,000 I.U, e.g. monthly will contribute to defining an algorithm that predicts the optimal supplementation intervals. However, as in particular in the elderly the oral intake of drugs might be diminished and/or in the presence of intestinal malabsorption disorders this route might be preferred.

Still, only limited data are available on the effects of oral and i.m. vitamin D supplementation and their pharmacokinetics. In a previous report, serum 25(OH)D levels gradually increased in 7 weeks after injection [26]. Here, after 100,000 I.U. i.m. the serum 25(OH)D concentrations peaked after 4 weeks and decreased in the following 4 weeks. The oral supplementation resulted in a rapid increase in serum 25(OH)D levels, which peaked about 1 week after first daily dose of 80 μg/kg (3,200 I.U./kg) cholecalciferol [26]. In comparison to i.m. injection, the increase in serum 25(OH)D levels were more rapid but also more transient after oral administration of vitamin D, as previously observed [27]. The sustained levels of serum 25(OH)D concentrations can be explained by the vitamin D fat tissue storage capacity with a slow and gradual release of i.m. administered cholecalciferol [26]. Interestingly, we observed the maximal increase of 25(OH)D in oral and i.m. group on day 28 upon supplementation. However, the mean 25(OH)D concentration increase in both groups was comparable. Our study shows some limitations. First, the open label protocol for oral vitamin D supplementation. Additionally, the study population included mostly young subjects. Larger cohorts considering elderly subjects should be analyzed in further randomized clinical trials.

In summary, our results confirm that the oral route of cholecalciferol administration rapidly increases 25(OH)D serum levels. However, discontinuing oral supplementation is followed by an early and more rapid decrease of 25(OH)D in contrast to the i.m. application.

## Conclusions

Administration of vitamin D either by oral or i.m. increased the 25(OH)D serum concentrations. Thus, both supplementation routes are relevant in clinical practice. As the pharmacokinetics depending on the administration route, different treatment protocols may be required. The data from this study and previous data suggest an oral cholecalciferol supplementation of 2,000 I.U.– 4,000 I.U. daily from January till March in healthy adults is sufficient to achieve adequate serum 25(OH)D status. Alternatively, 100,000 I.U. may be administered i.m. completely free from side effects in individuals with e.g. impaired- gastrointestinal resorption or limited compliance.

## Supporting Information

S1 File Dose escalation 25(OH) levels. (PPT)

S2 File Vitamin D levels und kinetik flow chart. (PPT)

S3 File ViDImmun protocol. (DOC)

S4 File Consort checklist. (DOC)
