# Supplementary material for: Pharmacokinetic Evaluation of a Single Intramuscular High Dose versus an Oral Long-Term Supplementation of Cholecalciferol
Source: PLoS One. 2017 Jan 23;12(1):e0169620. doi: 10.1371/journal.pone.0169620 (PMC5256876; doi:10.1371/journal.pone.0169620)
Supplement: S1 File — (PPT) [file pone.0169620.s001.ppt]

## Slide 1
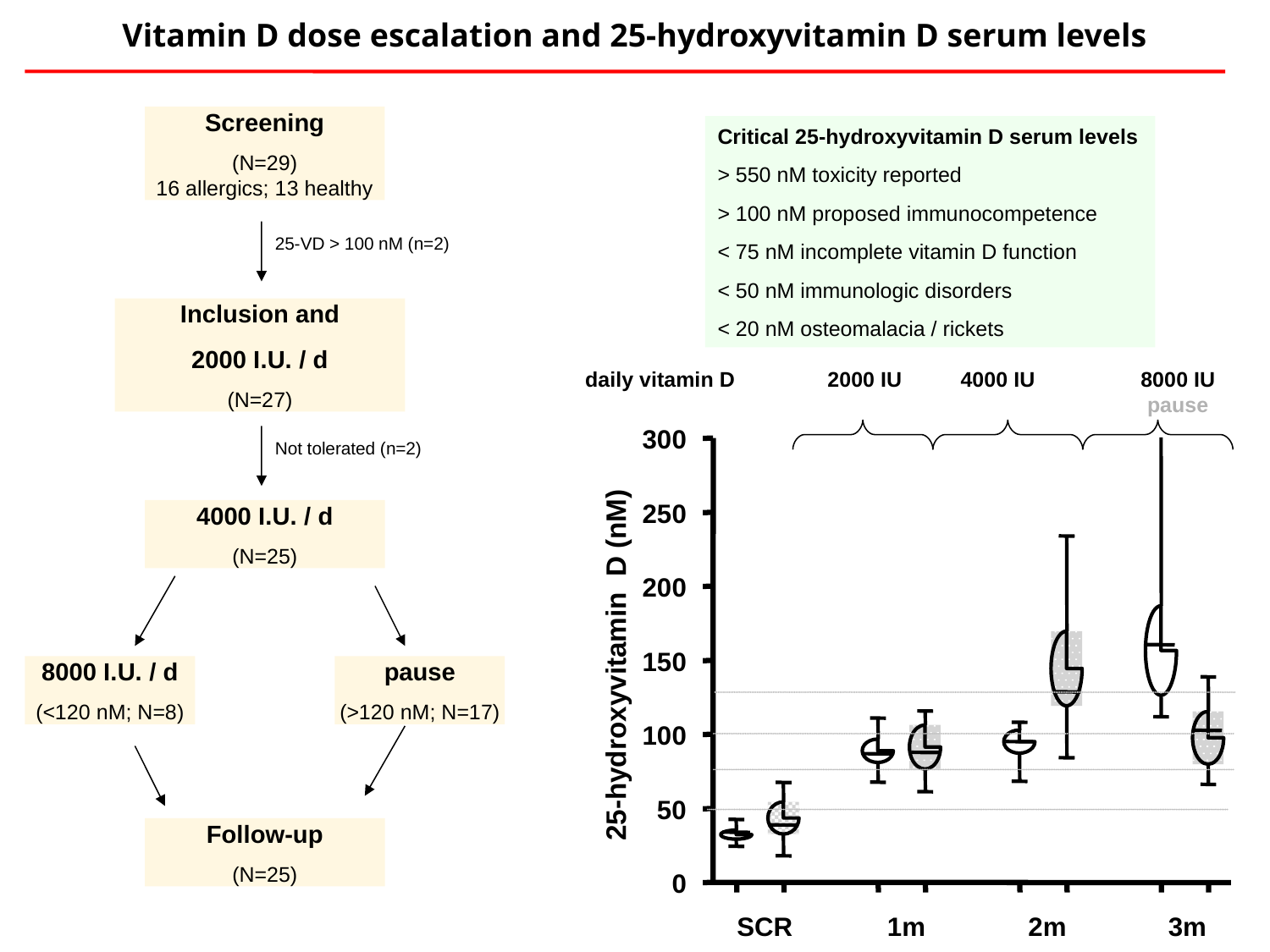

Vitamin D dose escalation and 25-hydroxyvitamin D serum levels
Screening
(N=29)
16 allergics; 13 healthy
Critical 25-hydroxyvitamin D serum levels
> 550 nM toxicity reported
> 100 nM proposed immunocompetence
< 75 nM incomplete vitamin D function
< 50 nM immunologic disorders
< 20 nM osteomalacia / rickets
25-VD > 100 nM (n=2)
Inclusion and
2000 I.U. / d
(N=27)
daily vitamin D
2000 IU
4000 IU
8000 IU pause
300
Not tolerated (n=2)
250
4000 I.U. / d
(N=25)
200
150
25-hydroxyvitamin D (nM)
8000 I.U. / d
(<120 nM; N=8)
pause
(>120 nM; N=17)
100
50
Follow-up
(N=25)
0
SCR
 1m
 2m
 3m
