# Supplementary material for: Pharmacokinetic Evaluation of a Single Intramuscular High Dose versus an Oral Long-Term Supplementation of Cholecalciferol
Source: PLoS One. 2017 Jan 23;12(1):e0169620. doi: 10.1371/journal.pone.0169620 (PMC5256876; doi:10.1371/journal.pone.0169620)
Supplement: S2 File — (PPT) [file pone.0169620.s002.ppt]

## Slide 1
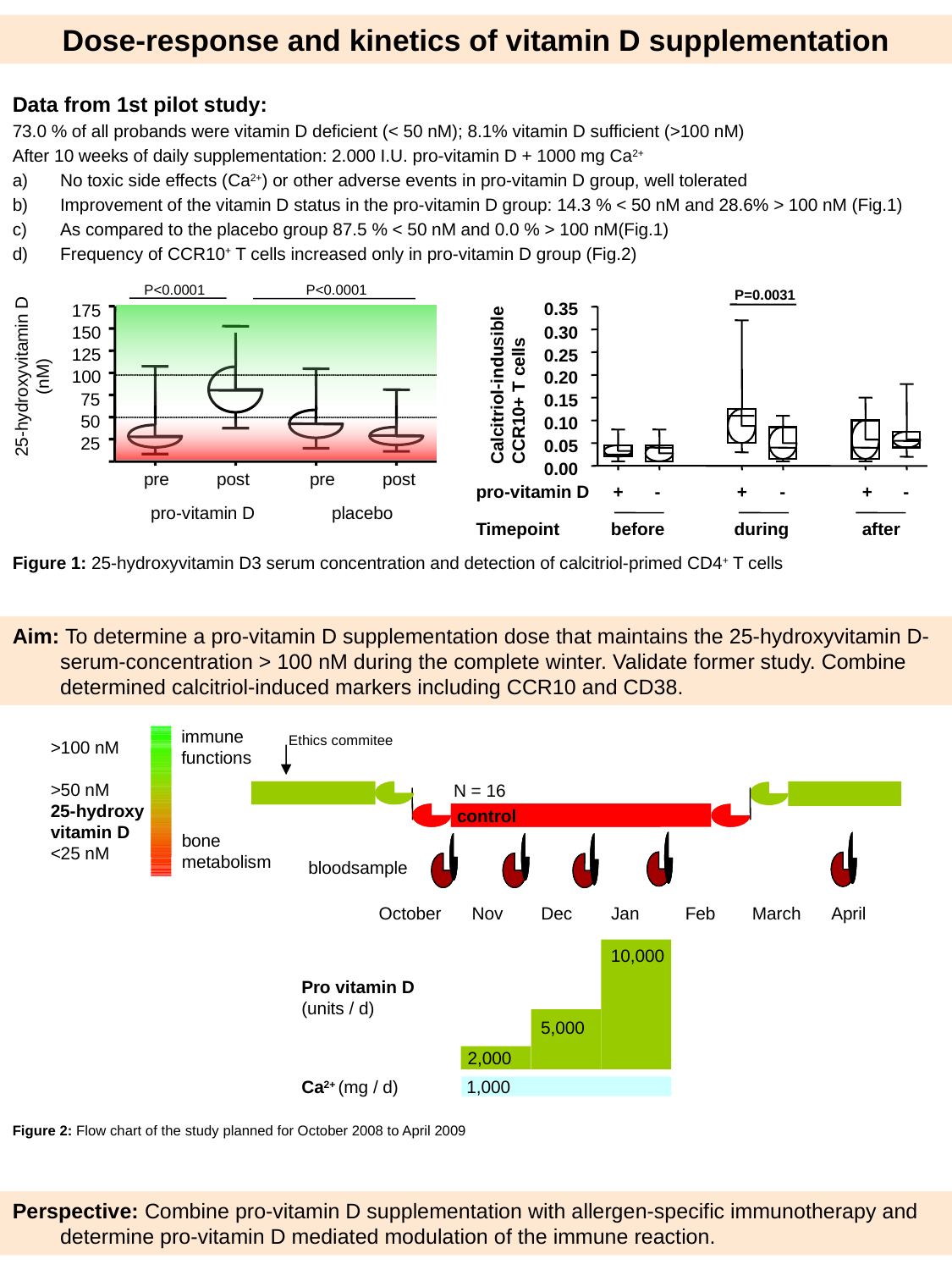

Dose-response and kinetics of vitamin D supplementation
Data from 1st pilot study:
73.0 % of all probands were vitamin D deficient (< 50 nM); 8.1% vitamin D sufficient (>100 nM)
After 10 weeks of daily supplementation: 2.000 I.U. pro-vitamin D + 1000 mg Ca2+
No toxic side effects (Ca2+) or other adverse events in pro-vitamin D group, well tolerated
Improvement of the vitamin D status in the pro-vitamin D group: 14.3 % < 50 nM and 28.6% > 100 nM (Fig.1)
As compared to the placebo group 87.5 % < 50 nM and 0.0 % > 100 nM(Fig.1)
Frequency of CCR10+ T cells increased only in pro-vitamin D group (Fig.2)
P<0.0001
P<0.0001
P=0.0031
0.35
0.30
0.25
Calcitriol-indusible
CCR10+ T cells
0.20
0.15
0.10
0.05
0.00
pro-vitamin D	+	 -	+	 -	+	 -
Timepoint	 before	 during	after
175
150
125
25-hydroxyvitamin D
(nM)
100
75
50
25
pre
post
pre
post
pro-vitamin D
placebo
Figure 1: 25-hydroxyvitamin D3 serum concentration and detection of calcitriol-primed CD4+ T cells
Aim: To determine a pro-vitamin D supplementation dose that maintains the 25-hydroxyvitamin D-serum-concentration > 100 nM during the complete winter. Validate former study. Combine determined calcitriol-induced markers including CCR10 and CD38.
immune
functions
>100 nM
>50 nM
25-hydroxy
vitamin D
<25 nM
control
control
bone
metabolism
bloodsample
October
Nov
Dec
Jan
Feb
March
April
10,000
5,000
2,000
Pro vitamin D
(units / d)
Ca2+ (mg / d)
1,000
Ethics commitee
N = 16
Figure 2: Flow chart of the study planned for October 2008 to April 2009
Perspective: Combine pro-vitamin D supplementation with allergen-specific immunotherapy and determine pro-vitamin D mediated modulation of the immune reaction.
