# Supplementary material for: Pharmacokinetic Evaluation of a Single Intramuscular High Dose versus an Oral Long-Term Supplementation of Cholecalciferol
Source: PLoS One. 2017 Jan 23;12(1):e0169620. doi: 10.1371/journal.pone.0169620 (PMC5256876; doi:10.1371/journal.pone.0169620)
Supplement: S3 File — (DOC) [file pone.0169620.s003.doc]

**Immunologische Wirkungen einer Einmalgabe von 100.000 I.E. Cholecalciferol (Vitamin D3)**

Kurzbezeichnung / Protokoll-Code: **ViDImmun**

EudraCT Nummer: 2012-003217-33

**Protokoll**

**Version 1.4 / Datum: 28.01.2013**

**Sponsor:** Charité-Universitätsmedizin Berlin

**Bevollmächtigte Vertreterin des Sponsors
und Leiterin der klinischen Prüfung:** Prof. Dr. med. Margitta Worm

___________________________________________________________________

**Der Sponsor, bevollmächtigte Vertreterin des Sponsors stimmt den Inhalten dieses Protokolls durch ihre Unterschrift zu und bestätigen, dass ihr die ICH-GCP-Richtlinie, die Anforderungen des AMG und der GCP-V bekannt sind und dass die klinische Prüfung nach diesen Vorschriften durchgeführt wird.**

| **Bevollmächtigte Vertreterin des Sponsors und Leiterin der klinischen Prüfung** |  |  |  |  |
| --- | --- | --- | --- | --- |
|  |  | Prof. Dr. med. M. Worm |  | Ort, Datum |
|  |  |  |  |  |

- Vertraulich -

Die Informationen in diesem Protokoll sind streng vertraulich zu behandeln. Sie dienen nur zur Information des Sponsors, seiner Mitarbeiter sowie beteiligten Prüfärzten und seiner projektbeteiligten Kooperationspartnern, der Ethik-Kommission des Landes Berlin und dem Bundesinstitut für Arzneimittel und Medizinprodukte (BfArM).

**Protokoll – Prüfung und Autoren**

**Bevollmächtigte Vertreterin** Prof. Dr. med. Margitta Worm **des Sponsors/ LKP und Autor:** Charité-Universitätsmedizin Berlin

Klinik für Dermatologie, Venerologie und Allergologie

Allergie-Centrum-Charité (ACC)

Charitéplatz 1, 10117 Berlin

Tel.: 030-450 518 105

Fax: 030-450 518 931

margitta.worm@charite.de

**Prüfarzt und Autor:** Dr. med. Guido Heine

Charité – Universitätsmedizin Berlin

Klinik für Dermatologie, Venerologie und Allergologie

Allergie-Centrum-Charité (ACC)

Charitéplatz 1, 10117 Berlin

Tel.: +49 (0)30-450 618 305

Fax: +49 (0)30-450 518 968

guido.heine@charite.de

**Projektkoordinatorin und Autor:** Dr. rer. medic. Sabine Dölle

Charité – Universitätsmedizin Berlin

Klinik für Dermatologie, Venerologie und Allergologie

Allergie-Centrum-Charité (ACC)

Charitéplatz 1, 10117 Berlin

Tel.: +49 (0)30-450 518 367

Fax: +49 (0)30-450 518 968

sabine.doelle@charite.de

***Inhaltsverzeichnis***

[1 Synopsis 7](#__RefHeading___Toc340700500)

[2 Ablaufschema 12](#__RefHeading___Toc340700501)

[3 Einleitung 13](#__RefHeading___Toc340700502)

[3.1 Einleitung und Hintergrund 13](#__RefHeading___Toc340700503)

[3.1.1 Vitamin D3 als immunmodulatorisches Hormon 13](#__RefHeading___Toc340700504)

[3.1.2 Bestimmung spezifischer Vitamin D3-vermittelter Wirkung auf Immunzellen 14](#__RefHeading___Toc340700505)

[3.2 Stand des Wissens über die Studienmedikation 16](#__RefHeading___Toc340700506)

[3.3 Fragestellung und Begründung des Vorhabens (Rationale) 16](#__RefHeading___Toc340700507)

[4 Ziel der klinischen Prüfung 17](#__RefHeading___Toc340700508)

[4.1 Hauptziel (*Objective*) 17](#__RefHeading___Toc340700509)

[4.2 Endpunkte 17](#__RefHeading___Toc340700510)

[4.3 Studiendesign 18](#__RefHeading___Toc340700511)

[4.4 Zeitplan 18](#__RefHeading___Toc340700512)

[5 Auswahl der Probanden 18](#__RefHeading___Toc340700513)

[5.1 Einschlusskriterien 18](#__RefHeading___Toc340700514)

[5.2 Ausschlusskriterien 18](#__RefHeading___Toc340700515)

[5.3 Nicht erlaubte Medikamente 19](#__RefHeading___Toc340700516)

[6 Behandlungsplan 19](#__RefHeading___Toc340700517)

[6.1 Studienmedikation 19](#__RefHeading___Toc340700518)

[6.1.1 Allgemeine Information zu D3-Vicotrat® 19](#__RefHeading___Toc340700519)

[6.1.2 Beschreibung der Studienmedikation 20](#__RefHeading___Toc340700520)

[6.1.3 Äußere Form der Studienmedikation 20](#__RefHeading___Toc340700521)

[6.1.4 Etikettierung 21](#__RefHeading___Toc340700522)

[6.2 Behandlungsschema 21](#__RefHeading___Toc340700523)

[6.2.1 Dosierung und Anwendung 21](#__RefHeading___Toc340700524)

[6.2.2 Bereitstellung der Studienmedikation und Zuordnung zu den Behandlungsgruppen 21](#__RefHeading___Toc340700525)

[6.2.3 Lagerung, Aus- und Rückgabe 21](#__RefHeading___Toc340700526)

[6.2.4 Verzeichnis der Nebenwirkungen und Wechselwirkungen 22](#__RefHeading___Toc340700527)

[6.2.5 Compliance 22](#__RefHeading___Toc340700528)

[6.3 Begleitmedikation / Begleittherapie 22](#__RefHeading___Toc340700529)

[6.4 Notfallmaßnahmen 23](#__RefHeading___Toc340700530)

[6.5 Randomisierung / Dekodierung 23](#__RefHeading___Toc340700531)

[6.5.1 Randomisierungsumschlag 23](#__RefHeading___Toc340700532)

[6.5.2 Notfallumschlag 23](#__RefHeading___Toc340700533)

[6.5.3 Reguläre Dekodierung nach Abschluss der klinisch wissenschaftlichen Untersuchung 24](#__RefHeading___Toc340700534)

[6.6 Randomisierung 24](#__RefHeading___Toc340700535)

[7 Studienablauf 24](#__RefHeading___Toc340700536)

[7.1 Verfahren der Rekrutierung / des Screenings 24](#__RefHeading___Toc340700537)

[7.2 Verfahren der Einwilligung nach Aufklärung 24](#__RefHeading___Toc340700538)

[7.3 Verfahren zur Vermeidung von zeitgleichem Einschluss in mehreren Studien 25](#__RefHeading___Toc340700539)

[7.4 Aufnahme und Registrierung 25](#__RefHeading___Toc340700540)

[7.5 Klinische Untersuchungen und Abweichungen von der üblichen klinischen Praxis 25](#__RefHeading___Toc340700541)

[7.5.1 Eingangs- / Einschlussuntersuchung 25](#__RefHeading___Toc340700542)

[7.5.2 Visite 1 (Randomisierung und Behandlungsstart) 25](#__RefHeading___Toc340700543)

[7.5.3 Visiten 2 (1 Tag), V3 (2 Tage) und V4 (3 Tage) nach Applikation der Studienmedikation) 26](#__RefHeading___Toc340700544)

[7.5.4 Visite 5 (7 ±1 Tage nach Applikation der Studienmedikation) 26](#__RefHeading___Toc340700545)

[7.5.5 Visite 6, 7, 8, 9 und 10 26](#__RefHeading___Toc340700546)

[7.5.6 Visite 11 (4 Wochen nach Applikation der Studienmedikation) 26](#__RefHeading___Toc340700547)

[7.5.7 Visite 12 (8 Wochen nach Applikation der Studienmedikation) 26](#__RefHeading___Toc340700549)

[7.5.8 Visite 13 (12 Wochen nach Applikation der Studienmedikation, Abschlussvisite) 26](#__RefHeading___Toc340700550)

[7.5.9 Laboruntersuchungen 27](#__RefHeading___Toc340700551)

[7.6 Beurteilung der Wirksamkeit 27](#__RefHeading___Toc340700552)

[Immunologische Wirksamkeit 27](#__RefHeading___Toc340700553)

[7.7 Sicherheit und Verträglichkeit 28](#__RefHeading___Toc340700554)

[7.7.1 Anamnese 28](#__RefHeading___Toc340700555)

[7.7.2 Körperliche Untersuchung und Vitalparameter 28](#__RefHeading___Toc340700556)

[7.7.3 Unerwünschte Ereignisse 28](#__RefHeading___Toc340700557)

[7.7.4 Sicherheitslabor 28](#__RefHeading___Toc340700558)

[7.7.5 Verträglichkeit 29](#__RefHeading___Toc340700559)

[7.7.6 Schwangerschaftstest und Kontrazeption 29](#__RefHeading___Toc340700560)

[7.8 Dauer der Teilnahme an der klinisch wissenschaftlichen Untersuchung für den einzelnen Probanden 29](#__RefHeading___Toc340700561)

[8 Risiko-Nutzen-Abwägung 29](#__RefHeading___Toc340700562)

[9 Abbruch und Weiterbehandlung 30](#__RefHeading___Toc340700563)

[9.1 Vorzeitiger Studienabbruch eines einzelnen Probanden 30](#__RefHeading___Toc340700564)

[9.2 Vorzeitiger Abbruch der gesamten klinischen Prüfung 31](#__RefHeading___Toc340700565)

[9.3 Plan für die Weiterbehandlung/medizinische Betreuung 31](#__RefHeading___Toc340700566)

[10 Unerwünschte Ereignisse 31](#__RefHeading___Toc340700567)

[10.1 Definitionen (nach Richtlinie 2001/20/EG) 31](#__RefHeading___Toc340700568)

[10.2 Beurteilung der Intensität 32](#__RefHeading___Toc340700569)

[10.3 Beurteilung des Kausalzusammenhanges 32](#__RefHeading___Toc340700570)

[10.4 Dokumentation von AEs und SAEs 33](#__RefHeading___Toc340700571)

[10.5 Meldung von SAEs und von Verdachtsfällen schwerwiegender unerwarteter unerwünschter Nebenwirkungen (SUSARs) 33](#__RefHeading___Toc340700572)

[10.6 Schwangerschaften 34](#__RefHeading___Toc340700573)

[11 Dokumentation 35](#__RefHeading___Toc340700574)

[11.1 Dokumentationsbögen (CRF) 35](#__RefHeading___Toc340700575)

[11.2 Prüfarztordner (ISF) 35](#__RefHeading___Toc340700576)

[11.3 Dokumentation der Studienmedikation (*Drug Accountability*) 35](#__RefHeading___Toc340700577)

[12 Qualitätsmanagement 35](#__RefHeading___Toc340700578)

[13 Dateneingabe und Datenmanagement 35](#__RefHeading___Toc340700579)

[13.1 Allgemeines 35](#__RefHeading___Toc340700580)

[13.2 Datenerhebung / Dokumentationsbögen 36](#__RefHeading___Toc340700581)

[13.3 Datenverarbeitung 36](#__RefHeading___Toc340700582)

[14 Statistische Analyse 37](#__RefHeading___Toc340700583)

[14.1 Fallzahlschätzung 37](#__RefHeading___Toc340700584)

[14.2 Statistische Auswertung 38](#__RefHeading___Toc340700585)

[15 Berichterstattung 40](#__RefHeading___Toc340700586)

[16 Publikation 40](#__RefHeading___Toc340700587)

[17 Ethische, rechtliche und verwaltungstechnische Aspekte 40](#__RefHeading___Toc340700588)

[17.1 Rechtliche Voraussetzungen für die Studie 40](#__RefHeading___Toc340700589)

[17.2 Aufbewahrung der Daten und Zugang zu den Daten 41](#__RefHeading___Toc340700590)

[18 Literaturverzeichnis 42](#__RefHeading___Toc340700591)

[Anhänge 42](#__RefHeading___Toc340700592)

**Abkürzungsverzeichnis**

AE *Adverse Event* = unerwünschtes Ereignis

ALT Alanin-Aminotransferase

AMG Arzneimittelgesetz

APZ antigenpräsentierende Zelle

BfArM Bundesinstitut für Arzneimittel und Medizinprodukte

BMI *Body mass index*

Calcitriol 1,25-Dihydroxyvitamin D

CCR *Chemokine Receptor*

CD C*luster of Differentiation*

CLA *cutaneous lymphocyte antigen*

CMV Cytomegalievirus

CRF *Case Report Form* = Dokumentationsbogen

CrTh2 *Chemoattractant receptor-homologous molecule expressed on Th2 lymphocytes*

CYP Cytochrom P (Enzyme: CYP27B1 und CYP24A1)

Diff-BB differential Blutbild

ESRD *End-stage-renal-disease*

GCP *Good Clinical Practice* = Gute Klinische Praxis

GCP-V Verordnung über die Anwendung der Guten klinischen Praxis bei der Durchführung von klinischen Prüfungen mit Arzneimitteln zur Anwendung am Menschen

GGT Gamma-Glutamyltransferase

HHV Humanes Herpesvirus

25-OH-VD 25-Hydroxyvitamin D (Provitamin D)

ICH *International Conference of Harmonisation*

IFN Interferon

Ig Immunglobulin

IIT *Investigator Initiated Trial*

IL Interleukin

i.m. intramuskulär

ISF *Investigator’s Site File*

LKP Leiterin der klinischen Prüfung

MFI *Mean Fluorescence Intensity*

MHC II *Major Histocompatibility Complex II* = Haupt-Histokompatibilitäts-Komplex II

NaCl Natriumchlorid

SAE S*erious Adverse Event* = schwerwiegendes unerwünschtes Ereignis

s.c. subkutan

SOP *Standard operating procedure*

*Source Data* Originaldokumente

SPSS *Statistical Package for the Social Sciences*

Th *T helper cell,* T-Helferzelle

TLR *toll-like receptor*

TMF *Trial Master File*

VDR Vitamin D Rezeptor

# Synopsis

| **Titel der Studie** | Immunologische Wirkungen einer Einmalgabe von 100.000 I.E. Cholecalciferol (Vitamin D3). |
| --- | --- |
| **Kurztitel** | ViDImmun |
| **Art des Vorhabens** | Explorative wissenschaftliche Untersuchung  Pilotprojekt  *Investigator Initiated Trial* (IIT) |
| **Sponsor** | Charité-Universitätsmedizin Berlin |
| **Bevollmächtigte Vertreterin des Sponsors/ Leiterin der klinischen Prüfung (LKP)** | Prof. Dr. med. Margitta Worm  Charité-Universitätsmedizin Berlin  Allergie-Centrum-Charité (ACC)  Klinik für Dermatologie, Venerologie und Allergologie  Charitéplatz 1, 10117 Berlin  Tel.: 030-450 518 105  Fax: 030-450 518 931  margitta.worm@charite.de |
| **Hypothese** | Es wird vermutet, dass durch die Gabe von Vitamin D3 (Cholecalciferol) intramuskulär (i.m.) oder subkutan (s.c.) eine immunologisch wirksame Provitamin D (25-Hydroxyvitamin D) Serumkonzentration erreicht wird, die in der Folge eine Modulation von Immunzellen ermöglicht. |
| **Hauptziel (*Objective*)** | Bestimmung des Anteils von Vitamin D3-reagierenden Zellen (CD38^+^ B-Zellen) im Blut im vorher-nachher Vergleich nach einmaliger i.m. bzw. s.c. Applikation bei Probanden mit Vitamin D3 Mangel. |
| **Fragestellung aufbauend auf wissenschaftlichem Hintergrund (Kap. 3.3)** | Diese klinisch wissenschaftliche Untersuchung soll die Frage klären, welche immunologischen Wirkungen durch Vitamin D3 vermittelt werden. Folgende Fragen werden bearbeitet:   - In welcher Häufigkeit zirkulieren Vitamin D3-reagierende Immunzellen im Blut? - Welche charakteristischen Funktionsparameter weisen diese Zellen auf? - Welchen Einfluss hat Vitamin D3 auf die humorale Immunität? |
| **Studienmedikation / Behandlungsstrategie (Kap. 6.1.2)** | Vitamin D3:  D3-Vicotrat®, Wirkstoff: Cholecalciferol (Vitamin D3), 1 Ampulle mit 1 ml Injektionslösung enthält: 2,5 mg Cholecalciferol (entsprechen 100.000 I.E. Vitamin D3)  Einmalige Gabe intramuskulär oder subkutan |
| **Vergleichsmedikation**  **(Kap. 6.1.2)** | Placebo:  Isotone Natriumchloridlösung (NaCl) 0,9% Injektionslösung; Inhaltsstoff: 100 ml Lösung enthalten NaCl 0,9 g und Wasser für Injektionszwecke  Einmalige Gabe intramuskulär oder subkutan |
| **Studiendesign (Kap. 4.3)** | Prospektiv, randomisiert, placebo-kontrolliert, doppel-blind, vier-armig (2 Applikationswege), explorativ |
| **Zeitplan (Kap. 4.4)** | Beginn der Rekrutierung: sobald Genehmigungen vorliegen  Screening-/Randomisierungsphase: sobald Genehmigungen vorliegen – 15.02.2013  Untersuchungsphase: 12 Wochen  Studienende: letzte Visite (V13) letzter Proband, spätestens 10.05.2013  Datenauswertung: 30.07.2013  Abschlussbericht: bis spätestens 1 Jahr nach Studienende |
| **Gesamtzahl Probanden** | Gesamt: 40 Probanden (incl. 4 Drop-outs) verteilt auf 4 Gruppen  Verum i.m. n=12 +1 Drop-out  Verum s.c. n=12 +1 Drop-out  Placebo i.m. n=6 + 1 Drop-out  Placebo s.c. n=6 + 1 Drop-out |
| **Studienpopulation** | Probanden mit Vitamin D3 Mangel, definiert durch ≤50 nmol/l (≤20 ng/ml)[[1]](#footnote-2) 25-Hydroxyvitamin D (25-OH-VD) im Serum. |
| **Einschlusskriterien (Kap. 5.1)** | - schriftliche Einwilligungserklärung - Männer und Frauen im Alter von 18 - 60 Jahren - 25-OH-VD Konzentration im Serum ≤50 nmol/l - bei Frauen im gebärfähigen Alter: Anwendung einer effektiven Kontrazeption |
| **Ausschlusskriterien  (Kap. 5.2)** | - 25-OH-VD Konzentration im Serum >50 nmol/l - BMI < 18,5 oder >30 kg/m² - Regelmäßige oder geplante UV-Exposition, z.B. Sonnenstudio >1x pro Woche, Reise an einen anderen Ort mit verstärkter UV-Exposition (UV-Index/Tag >5) - Überempfindlichkeit gegen einen der Bestandteile von D3-Vicotrat® oder Vergleichspräparat - Anamnestisch: Hyperkalzämie/-urie, Neigung zu Nierensteine, Niereninsuffizienz, Sarkoidose, Pseudohypoparathyreodismus[^1^](#_ENREF_1) - Personen mit hereditärer Fructose-Intoleranz - Einnahme von Glukokortikoiden - Behandlung mit Vitamin A-Derivaten oder anderen Vitamin D-haltigen Präparaten - Behandlung mit immunsuppressiv und/oder immunmodulatorisch wirksamen Medikamenten - Behandlung mit Phenytoin, Barbiturate, Thiazid-Diuretika, Herzglykoside - immobile Personen - Kalzium, Phosphat oder Kreatinin im Serum mit Werten außerhalb des entsprechenden Referenzbereiches (Anlage 6), klinisch relevante Abweichungen der hämatologischen Werte (Differenzialblutbild) - Unterbringung in einer Anstalt auf gerichtliche oder behördliche Anordnung - (geplante) Schwangerschaft und Stillzeit - bestehende Abhängigkeit zum Sponsor, bevollmächtigten Vertreterin des Sponsors, z.B. Verwandtschaft, Arbeitsverhältnis) - gleichzeitige Teilnahme an einer anderen Interventionsstudie innerhalb 30 Tage vor und während der hier geplanten Studie - sonstige Gründe, wie Drogen- und Alkoholabusus, zu erwartende fehlende Compliance |
| **Dokumentationszeitpunkte** | Screening  Visite 1 (Tag 1)  Visite 2 (Tag 2)  Visite 3 (Tag 3)  Visite 4 (Tag 4)  Visite 5 (Tag 7)  Visite 6 (Tag 14)  Visite 7 (Tag 21)  Visite 8 (Tag 28)  Visite 9 (Tag 35)  Visite 10 (Tag 42)  Visite 11 (Tag 49)  Visite 12 (Tag 56)  Visite 13 (Tag 84) |
| **Endpunkte**  **(Kap. 4.2)** | Primärer Endpunkt: Anteil im Blut zirkulierender CD38^+^ B-Zellen im vorher-nachher Vergleich bei Vitamin D3-Gabe (i.m. oder s.c.)  Sekundäre Endpunkte: Anteile von T- Zellen sowie myeloiden antigenpräsentierenden Zellen (APZs) im Blut, die klassische Vitamin D3-induzierte Oberflächenmarker aufweisen (CD38^+^CD4^+^ T-Zellen, HLA-DR^low^CD14^+^ Monozyten) vor und nach einer i.m. bzw. s.c. Injektion von Vitamin D3.   - weitere immunologische Parameter: - Veränderungen im T-Zell-Phänotyp und -Zytokinprofil  - Veränderungen im B-Zell-Phänotyp - verändertes Profil der Monozytenaktivierung - Veränderungen in der humoralen Immunantwort - Pharmakokinetik - Sicherheit - Verträglichkeit   Analyse des primären Endpunkts und der sekundären Endpunkte unter Berücksichtigung des Geschlechts. |
| **Sicherheit**  **(Kap. 7.7)** | - Anamnese - körperliche Untersuchung und Vitalparameter - Dokumentation (schwerwiegender) unerwünschter Ereignisse bei allen Visiten durch den Prüfarzt - Sicherheitslabor: Serum-Kalzium, Serum-Phosphat, Serum-Kreatinin, Alanin-Aminotransferase (ALT), Gamma-Glutamyltransferase (GGT), Differentialblutbild, Serum-25-OH-VD - Dokumentation der Verträglichkeit durch den Probanden und den Prüfarzt - Schwangerschaftstest |
| **Abbruchkriterien**  **(Kap. 9)** | - Hyperkalzämie - persönlicher Wunsch des Probanden - Eintritt einer Schwangerschaft - nicht ausreichende Compliance - Einnahme oder Anwendung von Medikamente, die ein Ausschlusskriterium darstellen - Jede Situation, die nach Ansicht des Prüfarztes einer weiteren Teilnahme an der klinisch wissenschaftlichen Untersuchung des Probanden entgegen sprechen würde. |
| **Statistische Auswertung**  **(Kap. 14.2)** | Geplante Analysen  Primärer Endpunkt:  Vergleich der Gruppen “Verum i.m. und s.c.“ mit der Gruppe “Placebo i.m./s.c.“ bezogen auf die vorher-nachher Differenz Vitamin D-reagierender CD38^+^ B-Zellen  Sekundäre Endpunkte:  Deskriptive Auswertung aller Variablen. Ferner Analyse des primären Endpunktes sowie der sekundären Endpunkte unter Berücksichtigung des Geschlechts. |
| **Pharmakologisch-toxikologische Prüfung** | D_3_-Vicotrat® ist seit fast 40 Jahren im Handel und ist das einzige, in Deutschland zugelassene Vitamin-D3-Präparat zur i.m. Applikation. Das Arzneimittel wird zur Prophylaxe von Vitamin-D-Mangelerscheinungen angewendet. Das pharmakologisch-toxikologische Gutachten der Fa. HEYL von 2008 liegt dem Antrag bei (Anhang 1). |
| **Mögliche Risiken, Nebenwirkungen, Kontraindikationen (Kap. 6.2.4)**  **Maßnahmen, die bei eventuellen Zwischenfällen zu ergreifen sind** | Nebenwirkungen im Rahmen einer Überdosierung - Hyperkalzämie (laut Fachinformation August 2009):  Akute Symptome: Herzrhythmusstörungen, Übelkeit, Erbrechen, psychische Symptome, Bewusstseinsstörungen  Chronische Symptome: vermehrter Harndrang, verstärktes Durstgefühl, Appetitlosigkeit, Gewichtsverlust, Nierensteinbildung, Nierenverkalkung, Verkalkung in Geweben außerhalb des Knochens. In Einzelfällen sind tödliche Verläufe (bei Überdosierung) beschrieben worden.  Bei Verdacht auf das Vorliegen von Vitamin D-vermittelter Toxizität liegt ein Notfallplan vor, der sofortige Serum-Kalzium, Serum-Phosphat, Serum-Kreatinin und Serum-25-OH-VD Messung vorsieht, kalziumarme Diät und ggf. i.v. stadiengerechte Gabe von isotonischer NaCl-Lsg. (3-6 l/ Tag) mit Zusatz von Furosemid sowie u.U. auch 15 mg/kg KG/Stunde Natriumedetat unter EKG/Kalzium-Kontrolle. Bei Oligoanurie ist eine Hämodialysetherapie indiziert.  Schwangerschaft  Bei diagnostizierter Schwangerschaft erfolgt sofortiger Studienabbruch (Kap. 10.6). Da lang anhaltende Überdosierungen von Vitamin D in der Schwangerschaft zu körperlicher und geistiger Retardierung, supravalvulärer Aortenstenose und Retinopathie des Kindes führen kann (Folgen der Hyperkalzämie).  Stillzeit  Aus medizinischen wie auch ethischen Gründen werden stillende Probanden nicht in die klinisch wissenschaftliche Untersuchung eingeschlossen.  Vitamin D und seine Stoffwechselprodukte gehen in die Muttermilch über. Eine auf diesem Wege erzeugte Über­dosierung beim Säugling ist nicht beobachtet worden.  Blutentnahmen  Die durchgeführten Blutentnahmen werden durch klinisch geschultes und ärztliches Personal durchgeführt und weichen nicht von den routinemäßigen Verfahren ab. |
| **Risiko-Nutzen-Abwägung**  **(Kap. 8)** | Es existieren keine Berichte über Vitamin D3-vermittelte Nebenwirkungen der im Rahmen dieser Studie einmaligen Gabe von 100.000 I.E. (Stand Mai 2012). Über nachteilige immunmodulatorische Wirkungen von Provitamin D (25-OH-VD) in den hier erwarteten Serumkonzentrationen (115 ±7,1 nmol/l) liegen keine Berichte vor. Im Gegenteil wird mit den hier verwandten Vitamin D3 Mengen der Vitamin D3 Mangel (Einschlusskriterium) ausgeglichen, was positiv ist und für den Knochenstoffwechsel empfohlen wird und auch für günstige immunologische Funktionen diskutiert wird.  Das Risiko einer Hyperkalzämie durch die Vitamin D3 Applikation ist als sehr gering einzustufen, da alle Probanden zu Beginn einen Vitamin D3 Mangel aufweisen. Durch zahlreiche Untersuchungen wurde gezeigt, dass die vorgesehene Dosierung als sicher einzustufen ist (siehe Kap. 6.2.1 und 6.2.4.).  Durch die Einmaldosis von 100.000 I.E. Vitamin D3 mittels i.m. bzw. s.c. Injektion können definierte Zielkonzentrationen von Provitamin D (25-OH-VD) erzielt werden, da keine Abhängigkeit zur Resorptionsrate im Darm besteht. Zudem kann im Gegensatz zur täglichen Einnahme eine 100%-ige Compliance erzielt werden und der Vitamin D3 Mangel schnell ausgeglichen werden.  Somit können auch Vitamin D3-reagierende Immunzellen im Blut günstig nachgewiesen werden, da sie gleichzeitig auf Vitamin D3 reagieren und nicht sequenziell, wie es bei einer täglichen oralen Einnahme der Fall wäre. |

# Ablaufschema

|  | **Screen** | **V1** | **V2** | **V3** | **V4** | **V5** | **V6** | **V7** | **V8** | **V9** | **V10** | **V11** | **V12** | **V13** |
| --- | --- | --- | --- | --- | --- | --- | --- | --- | --- | --- | --- | --- | --- | --- |
| Tage (T), Wochen (W) | bis -W4 | T1 | T2 | T3 | T4 | T7±1T | T14±1T | T21±1T | T28±1T | T35±1T | T42±1T | T49±1T | T56±1T | T84±2T |
| Probandenaufklärung | ✓ |  |  |  |  |  |  |  |  |  |  |  |  |  |
| Einwilligungserklärung | ✓ |  |  |  |  |  |  |  |  |  |  |  |  |  |
| Anamnese | ✓ |  |  |  |  |  |  |  |  |  |  |  |  |  |
| Einschlusskriterien | ✓ | ✓ |  |  |  |  |  |  |  |  |  |  |  |  |
| Ausschlusskriterien | ✓ | ✓ |  |  |  |  |  |  |  |  |  |  |  |  |
| Randomisierung |  | ✓ |  |  |  |  |  |  |  |  |  |  |  |  |
| Körpergröße/-gewicht | ✓ |  |  |  |  |  |  |  |  |  |  |  |  |  |
| Körperl. Untersuchung | ✓ |  |  |  |  |  |  |  |  |  |  |  |  |  |
| Vital-Parameter^#^ | ✓ | ✓ |  |  |  |  |  |  | ✓ |  |  |  | ✓ | ✓ |
| Schwangerschaftstest^##^ | ✓ |  |  |  |  |  |  |  |  |  |  |  |  |  |
| Sicherheitslabor^###^ | ✓ |  |  |  |  |  |  |  | ✓ |  |  |  | ✓ | ✓ |
| Pharmakokinetik | ✓* | ✓** | ✓ | ✓ | ✓ | ✓ | ✓ | ✓ | ✓ | ✓ | ✓ | ✓ | ✓ | ✓ |
| BE Immunologie |  | ✓ |  |  |  | ✓ 200 ml° |  |  | ✓ 200 ml° |  |  |  |  | ✓ |
| Applikation der  Studienmedikation |  | ✓ |  |  |  |  |  |  |  |  |  |  |  |  |
| Verträglichkeit der Studienmedikation |  | ✓ |  |  |  |  |  |  |  |  |  |  |  |  |
| Erfassung unerwünschten Ereignissen (AE) |  | ✓ | ✓ | ✓ | ✓ | ✓ | ✓ | ✓ | ✓ | ✓ | ✓ | ✓ | ✓ | ✓ |
| Medikamentenabfrage | ✓ | ✓ | ✓ | ✓ | ✓ | ✓ | ✓ | ✓ | ✓ | ✓ | ✓ | ✓ | ✓ | ✓ |

BE – Blutentnahme, FU – Follow-up, V – Visite

^#^ Vitalparameter umfassen Blutdruck und Puls

^##^ bei gebärfähigen Frauen

^###^Sicherheitslabor umfasst Kalzium, Phosphat, Kreatinin, Alanin-Aminotransferase (ALT), Gamma-Glutamyltransferase (GGT), Differential-Blutbild (Diff-BB)

* Hier erfolgt nur die die Bestimmung von 25-Hydroxyvitamin D (25-OH-VD).

** Pharmakokinetik wird vor und 1h, 2h und 6h nach Applikation der Studienmedikation gemessen.

° nur bei männlichen Probanden erfolgen 200 ml (Hb > 16,0 g/dl Hb)

# Einleitung

## Einleitung und Hintergrund

### Vitamin D3 als immunmodulatorisches Hormon

Vitamin D3 vermittelt immunologische Funktionen durch direkte Wirkung in Immunzellen, jedoch ist über die exakten Zielzellen und deren Modulation wenig bekannt.

Physiologisch wird Vitamin D3 zu 90-100% durch UV-Photobiosynthese aus 7-Dehydrocholesterol gebildet und ist biologisch nicht aktiv (Abb. 3-1) [^2^](#_ENREF_2). In Deutschland spielt direkt nutritionelles Vitamin D3, z.B. aus Lebertran, keine wesentlich Rolle [^3^](#_ENREF_3). Nach konsekutiver 25-Hydoxylierung in der Leber entsteht der inerte Speichermetabolit Provitamin D (chem. 25-Hydroxyvitamin D, 25-OH-VD), dessen Serumkonzentration der optimale Marker für den Vitamin D3 Status darstellt [^4^](#_ENREF_4). Der biologisch aktive Vitamin D3 Metabolit, Calcitriol (1,25-dihydroxyvitamin D), wird durch das streng transkriptionell regulierte Enzym Cytochrom P (CYP)27B1 zum größten Teil in der Niere gebildet. Calcitriol reguliert nach Bindung an den nukleären Vitamin D Rezeptor (VDR) die Aktivierung von Zielgenen, die spezifische Erkennungssequenzen in der Promotorregion besitzen. Die Inaktivierung von Calcitriol erfolgt in der Zielzelle durch das Enzym CYP24A1, das die 24-Hydroxylierung und somit die Synthese der wasserlöslichen Calcitroinsäure katalysiert, die über Gallensäuren, bzw. den Urin ausgeschieden wird (nicht gezeigt).

**Abbildung 3-1: Vitamin D3 Stoffwechsel und Biosynthese von Calcitriol.**

Immunmodulatorische Funktionen von Vitamin D3 sind durch epidemiologische und experimentelle Daten evident. Unterschiedliche Immunzellen können nach Aktivierung Calcitriol aus der inerten Vorstufe Provitamin D (25-OH-VD) synthetisieren, über den VDR detektieren und metabolisieren. Somit ist autokrines und parakrines Calcitriol-Signaling in diesen Zellen, z.B. nach antigenspezifischer Stimulation möglich. Das dafür notwendige Enzym CYP27B1 wurde in aktivierten myeloiden Immunzellen , T-Zellen [^7^](#_ENREF_7) funktionell gezeigt und Calcitriolsynthese gemessen. Eigene Daten belegen, dass auch aktivierte humane B-Zellen biologisch aktives Calcitriol aus der inerten Vorstufe synthetisieren können [^8^](#_ENREF_8), was mittlerweile bestätigt wurde [^9^](#_ENREF_9).

Direkte Wirkungen des aktivierten VDR auf humane Immunzellen werden mit Induktion von immunologischer Toleranz und Zellmigration diskutiert; im Detail:

CD4^+^ T-Helferzellen: Induktion von CD200 [^10^](#_ENREF_10), das tolerogene Zytokin Interleukin (IL)-10 [^11^](#_ENREF_11) [^12^](#_ENREF_12), *toll-like receptor-9* (TLR-9) [^13^](#_ENREF_13), dem Chemokinrezeptor CCR10 [^7^](#_ENREF_7), CD38 [^14^](#_ENREF_14). Hemmung von *cutaneous lymphocyte antigen* (CLA) [^15^](#_ENREF_15), sowie verminderte Ratio der proinflammatorischen Zytokine Interferon (IFN)-γ zu IL-4 [^12^](#_ENREF_12).

CD19^+^ B-Zellen: Induktion von CD38 [^14^](#_ENREF_14), IL-10 [^8^](#_ENREF_8) sowie in terminal differenzierten Plasmablasten zusätzlich CCR10 [^16^](#_ENREF_16).

Myeloide antigenpräsentierende Zellen (APZs): CD14^+^ Monozyten und myeloide dendritische Zellen): Differenzierung zu unreifen, tolerogenen dendritischen Zellen [^6^](#_ENREF_6)

Epidemiologische Daten deuten eine biologische Kontrollfunktion von Vitamin D3 während inflammatorischer Immunreaktionen an . Es wird ein kausaler Zusammenhang zwischen erworbenem Vitamin D3 Mangel, messbar an der Serumkonzentration von 25-OH-VD (Provitamin D) <50 nmol/l ^2^, mit immunologischen Krankheiten diskutiert. Dazu zählen multiple Sklerose [^19^](#_ENREF_20), rheumatoide Arthritis [^20-23^](#_ENREF_21), cutaner Lupus erythematodes ^24,25^ aber auch allergisches Asthma ^26,27^ oder atopische Dermatitis ^28,29^. Ein mögliches Risiko für die Entstehung von Allergien durch Vitamin D3 Mangel wird durch eine inverse Korrelation der 25-OH-VD Konzentration im Nabelschnurblut mit dem Immunglobulin (Ig)E-Spiegel angedeutet [^30^](#_ENREF_31). Eigene Arbeiten zeigen, dass der Isotypen­klassen­wechsel zu IgE und dessen Produktion durch direkte Wirkung von Calcitriol auf die B-Zellen gehemmt wird ^31,32^. Eine biologische Signifikanz dieser Beobachtung wird in folgenden präklinischen Modellen offensichtlich. So ist die spezifische IgE-Induktion vermindert durch Behandlung mit einem hypokalzämischen VDR-Agonisten [^33^](#_ENREF_34), sowie verstärkt in Mäusen mit defektem Vitamin D Stoffwechsel ([^34^](#_ENREF_35) sowie eigene unpublizierte Daten) oder defektem VDR ^34^.

### Bestimmung spezifischer Vitamin D3-vermittelter Wirkung auf Immunzellen

Für die therapeutische Aktivierung des Vitamin D Rezeptors (VDR) in Immunzellen wurde bislang die Verwendung synthetischer, niedrig-kalzämischer VDR-Agonisten angestrebt ^35^. Aufgrund toxischer Wirkungen dieser Substanzen in geringen Serumkonzentrationen ist deren Entwicklung nicht weiter vorangeschritten und derzeit sind keine synthetischen, immunmodulatorischen VDR-Agonisten für die Anwendung im Menschen zugelassen.

Die Verwendung von Calcitriol als VDR-Agonist ist aufgrund der kleinen therapeutischen Breite und hyperkalzämischen Wirkungen in Dauer und Stärke limitiert (Tabelle 3-1). Auch wenn durch Calcitriol-Gaben kurzfristig Vitamin D3-spezifische Wirkungen in Patienten mit allergischem Asthma messbar waren , so ist Calcitriol (aktives Vitamin D3) nicht im Zusammenhang mit einer langfristigen Behandlung vorstellbar, z.B. eines kutanen Lupus erythematodes oder einer mehrjährigen allergenspezifischen Immuntherapie.

**Tabelle 3-1: Pharmakologische Vorteile von Provitamin D (25-OH-VD) im Vergleich zu Calcitriol.**

Vitamin D3 Mangel (Provitamin D < 50 nmol/l) ist häufig und hat eine Prävalenz nach aktuellen Schätzungen und eigenen Daten in >50% der Durchschnittsbevölkerung im arbeitsfähigen Alter, insb. im Winter ^2,3,24^. Als Ursache wird reduzierte Sonnenexposition im Sommer aufgrund vermehrter Innenraumarbeit angenommen.

Da aktivierte Immunzellen aus exogenem Vitamin D3 selbst wirksames Calcitriol synthetisieren können, das autokrin und parakrin immunmodulatorisch wirkt, eröffnet sich durch Vitamin D3 Gabe die Möglichkeit Vitamin D3-spezifische Immunmodulation zu bestimmen. Dazu werden die Untersuchungen im UV-armen Winter durchgeführt, da im Vergleich der Behandlungsgruppe mit der Placebogruppe große Unterschiede in den 25-OH-VD (Provitamin D) Serumkonzentrationen auftreten werden, die mit den immunologischen Parametern assoziiert sind. Dafür sind hohe Vitamin D3 Dosierungen notwendig, z.B. durch orale Supplementation mit tgl. 20.000 I.E. oder 40.000 I.E. Vitamin D3 bei Patienten mit Multipler Sklerose oder mit 2x/Woche 50.000 I.E. bei Patienten mit end-stage-renal-disease (ESRD) [^37^](#_ENREF_37). Wir erwarten im Rahmen dieser Untersuchung mit einmaliger Gaben von 100.000 I.E. Vitamin D3 ebenfalls ausreichend hohe 25-OH-VD Serumkonzentrationen zu erreichen (115 ±7,1 nmol/l).

Aktivierung der Immunzellen ist notwendig für die Induktion von CYP27B1 und damit der Fähigkeit aus Provitamin D (25-OH-VD) das biologisch aktive Calcitriol zu synthetisieren. Aus Publikationen ist bekannt, dass viralen und bakteriellen Infektionen (z.B. durch Mykobakterium tuberkulosis getriggert[^1^](#_ENREF_1)) dies vermitteln können. In eigenen präklinischen Untersuchungen fanden wir heraus, dass auch Kontakt mit Allergenen (z.B. Pollen) CYP27B1 induziert. Im Rahmen dieser Untersuchung soll auf exogene Antigene zur Initiierung der spezifischen Immunantwort verzichtet werden. Es werden vielmehr spezifische Immunreaktionen gegen häufige, endogen vorkommende Antigene untersucht, wie z.B. Ebstein-Barr-Virus (Humanes Herpesvirus-4, HHV-4, Prävalenz in Deutschland >95%), Cytomegalievirus (HHV-5, Prävalenz in Deutschland bei Erwachsenen 30%), Varizella-Zoster-Virus (HHV-3, Prävalenz 90%). Dies erfolgt durch Bestimmung der spezifischen Antikörperkonzentrationen im Serum.

Zu den vermuteten Vitamin D3-vermittelten Wirkungen, die wir im Rahmen dieser Untersuchung messen möchten, zählt die Induktion von klassischen Vitamin D3-induzierbaren Oberflächenantigenen CD38 auf Lymphozyten und Hemmung von HLA-DR auf CD14^+^ Monozyten. Darüber hinaus gehen wir davon aus, dass sich Veränderungen im T-Zell-Zytokinprofil finden lassen, analog o.g. Publikationen eine verminderte Ratio der T-Zellhäufigkeiten die proinflammatorische Zytokine wie IL-17, IFN-γ und IL-4 exprimieren zu IL-10. Daraus resultierend könnten die sezernierten spezifischen anti-viralen Antikörper (z.B. Ebstein-Barr-Virus) in der Konzentration moduliert sein, analog publizierter Daten [^38^](#_ENREF_38).

Zusammenfassend legen die vorliegenden Daten nahe, dass durch Gabe von Vitamin D3 die notwendige Konzentration der Vorstufe Provitamin D (25-OH-VD) erreichbar ist, um endogene Calcitriolsynthese in Immunzellen zu ermöglichen was in Vitamin D3-spezifischer Immunmodulation resultiert. Die Analyse von T- und B-Zellen die für endogene, persistierende Antigenen spezifisch sind erlaubt durch die Kombination mit Vitamin D3-spezifischen Parametern die Bestimmung der Häufigkeit auf Vitamin D3-reagierender Zellen, die Dauer der Modulation nach Vitamin D3 Gabe sowie die Konsequenz für die humorale Immunreaktion.

## Stand des Wissens über die Studienmedikation

Die Fachinformation (August 2009) der Studienmedikation D3-Vicotrat® ist dem Antrag angefügt. Es ist für die Prophylaxe von Vitamin-D-Mangelerscheinungen bei Malabsorption zugelassen (Zulassungs-Nr. 6813051.00.00), wenn eine orale Therapie nicht möglich oder nicht wirksam ist.

In dieser klinisch wissenschaftlichen Untersuchung werden Probanden mit Vitamin D3 Mangel eingeschlossen. Eine orale Vitamin D3 Supplementation wäre grundsätzlich möglich, die Resorption kann jedoch individuell sehr unterschiedlich sein. Da wir die immunologischen Veränderungen untersuchen wollen, möchten wir durch die i.m. bzw. s.c. Applikation eine standardisierte und rasche Anflutung von Vitamin D3 erzielen.

Durch die geplante Bestimmung von Sicherheitsparametern (Kap. 7.7.4) und Vitamin D3 Metaboliten (25-OH-VD), wird die Sicherheit der Probanden engmaschig kontrolliert.

In diesem Zusammenhang möchten wir D3-Vicotrat® auch s.c. anwenden. Diese Darreichungsform ist aus historischen Gründen außerhalb der derzeitigen Zulassung. Jedoch sind die u.g. Bestandteile als solche, wie auch in der angewendeten Konzentration für die s.c. Anwendung geeignet und werden standardmäßig in anderen Präparationen verwandt (Natriumdihydrogenphosphat-Dihydrat, Natriumhydroxid und Polysorbat 80 z.B. in Abseamed®, Humira und Sorbitol/Sorbitollösung 70% in z.B. Simponi®, Ratiograstim).

Die Präparation enthält Vitamin D3 (Synonym: Cholecalciferol), analog dem natürlichen Metaboliten, der aus 7-Dehydrocholesterol nach UV-Strahlung entsteht und in der Leber zum inertem Vitamin D3 Speichermetaboliten 25-OH-VD (Provitamin D) verstoffwechselt wird.

Sonstigen Bestandteile sind Natriumdihydrogenphosphat-Dihydrat, Natriumhydroxid, Sorbitollösung 70 % (kristallisierend), Polysorbat 80, mittelkettige Triglyceride und Wasser für Injektionszwecke. D3-Vicotrat® ist zur i.m. Applikation zugelassen.

Durch die Applikation von D3-Vicotrat® wird der Speichermetabolit 25-OH-VD erhöht und damit der Vitamin D3 Status verbessert. Bei Erwachsenen mit normaler Funktion der Nebenschilddrüsen liegt die Schwelle für Vitamin D3 Intoxikationen zwischen 40.000 und 100.000 I.E. pro Tag über 1 bis 2 Monate. Zu den möglichen unerwünschten Wirkungen zählt im Wesentlichen die Hyperkalzämie, die durch Überdosierungen oder Begleiterkrankungen wie Sarkoidose, Niereninsuffizienz oder Pseudohypoparathyreoidismus entstehen kann (Kap. 6.2.4).

## Fragestellung und Begründung des Vorhabens (Rationale)

Diese doppel-blinde, placebo-kontrollierte, klinisch wissenschaftliche Untersuchung (Pilotstudie) soll ermöglichen immunologische Wirkungen von Vitamin D3 auf Immunzellen zu bestimmen. Folgende Fragen werden zentral bearbeitet:

- In welchem Anteil zirkulieren Vitamin D3-reagierende Immunzellen im Blut?
- Welche charakteristischen Funktionsparameter weisen diese Zellen auf?
- Welchen Einfluss hat Vitamin D3 auf die humorale Immunität?

In bisherigen Untersuchungen zu immunologischen Funktionen von Vitamin D3 wurden meist sehr geringe Vitamin D3 Dosierungen verwendet (1.000-2.000 I.E. tgl ). Auch war Vitamin D3 Defizienz kein Einschlusskriterium. In der Folge war die Wirkstärke auf definierte Zellpopulationen stark limitiert.

Die durchgeführten Untersuchungen von Zytokinkonzentrationen im Serum bzw. in Zellkulturen die unterschiedliche Unterpopulationen enthalten, geben keinen Aufschluss darüber welche und wie viele Zellen letztendlich die Veränderungen bewirkt haben.

Dies wäre wichtig um perspektivisch diese Zellen noch gezielter zu verändern. In neueren Untersuchungen wurden zwar ausreichend hohe Vitamin D3 Dosierungen verwandt (tgl. 20.000 I.E. oral), jedoch war die Analyse auf adaptive Immunzellen wie T-Lymphozyten begrenzt [^12^](#_ENREF_12).

In eigenen Untersuchungen führen wir bereits ein detailliertes Immunmonitoring durch, in dem Einzelzellanalysen, zum Erfassen der zellulären Immunantwort einschließlich der Häufigkeit Vitamin D-abhängiger Zellen, mit Parametern der humoralen Immunreaktion kombiniert werden (Studienkürzel ProGIT, EudraCT Nr. 2010-021775-80, registriert bei www.clinicaltrials.gov unter der Nummer: NCT01466465).

Jetzt wollen wir mit diesen Untersuchungen herausfinden, welche Zellpopulation durch autokrine Calcitriolsynthese, die durch Vitamin D3 Gabe an Vitamin D3-defiziente Probanden ermöglicht wird, hauptsächlich angesprochen wird.

Zu wissen, ob T- oder B-Lymphozyten oder myeloide APZs die Vitamin D3 Zielzellen sind, hat einen großen Einfluss auf die Entwicklung moderner Therapien. Beispielsweise wäre eine konstante Vitamin D3 Behandlung aussichtsreich, wenn Zellen des adaptiven Immunsystems (T- und B-Lymphozyten) moduliert werden. Wären die APZs die Zielzellen von Vitamin D3, dann könnte eine lokalisierte Vitamin D3 Therapie erfolgversprechend sein, z.B. mit Liposomen die Allergene und Vitamin D3 enthalten und somit Vitamin D3 nicht systemische wirkt.

# Ziel der klinischen Prüfung

## Hauptziel (*Objective*)

Ziel dieser Untersuchung ist die Bestimmung der Wirkung von Vitamin D3 (Cholecalciferol) auf die zelluläre und humorale Immunreaktion nach einmaliger i.m. bzw. s.c. Applikation bei Probanden mit Vitamin D3 Mangel.

## Endpunkte

Ziele dieser klinisch wissenschaftlichen Pilot-Untersuchung beinhalten

Primärer Endpunkt: Anteil im Blut zirkulierender CD38^+^ B-Zellen im vorher-nachher Vergleich (V1/V8) bei Vitamin D3-Gabe (i.m. oder s.c.)

Sekundäre Endpunkte:

- Anteil im Blut zirkulierender CD38^+^ B-Zellen im vorher-nachher Vergleich bei Vitamin D3-Gabe i.m., bzw. s.c. zu den übrigen Zeitpunkten

Vergleich vor und zu allen Untersuchungszeitpunkten nach einer i.m. bzw. s.c. Injektion von Vitamin D3:

- Anteile von T-Zellen und myeloiden antigenpräsentierenden Zellen (APZs) im Blut, die klassische Vitamin D3-induzierte Oberflächenmarker aufweisen (CD38^+^CD4^+^ T-Zellen, HLA-DR^low^CD14^+^ Monozyten)
- Veränderungen im T-Zell-Phänotyp (naive und Gedächtniszellen) sowie Zytokinprofil (Häufigkeit von IFN-γ, IL-17, IL-4 sowie IL-10 produzierender CD4^+^ T Helferzellen im Blut)
- Veränderungen im B-Zell-Phänotyp (naive und Gedächtniszellen)
- verändertes Profil der Monozytenaktivierung (Häufigkeit HLA-DR^low^CD14^+^ Monozyten sowie CD16^high^CD14^+^ im Blut)
- Veränderungen in der humoralen Immunantwort (anti-EBV-, anti-CMV-, anti-VZV-Immunglobulin im Serum)
- Pharmakokinetik (Vitamin D3 Metabolite im Serum)
- Sicherheit (Blutwerte: Ca, P, Krea, ALT, GGT, diff-BB.)
- Verträglichkeit (Visuelle Analogskala)

Die Auswertung des primären und der sekundären Endpunkte erfolgt ferner auch nach Geschlechterunterscheidung.

## Studiendesign

Probanden im Alter von 18 - 60 Jahren mit Vitamin D3 Mangel werden für diese prospektive, monozentrische, randomisierte, placebo-kontrollierte, doppel-blinde, vierarmige, explorative Untersuchung (Pilotprojekt) rekrutiert.

In einer Eingangsuntersuchung (Screening) wird die Eignung zur Studienteilnahme eingeschätzt. Es werden 40 Probanden eingeschlossen und zufällig einer der beiden Applikationswege i.m. oder s.c. zugeordnet. Innerhalb der jeweiligen Applikationsgruppe werden die Probanden im Verhältnis von 2:1 in die Vitamin D3- oder Placebogruppe randomisiert (Abb. 4-1).

**Abb. 4-1: Behandlungsgruppen**

## Zeitplan

Der Studienbeginn ist für den Dezember 2012 geplant, nach Vorliegen aller notwenigen Genehmigungen und den schriftlichen Einwilligungserklärungen der Probanden.

- Beginn der Rekrutierung: sobald Genehmigung vorliegend
- Screening-/Randomisierungsphase: sobald Genehmigung vorliegend – 15.02.2013
- Untersuchungsphase: 12 Wochen
- Studienende: letzte Visite (V13) letzter Proband, spätestens 10.05.2013
- Datenauswertung: 30.07.2013
- Abschlussbericht: bis spätestens 1 Jahr nach Studienende

# Auswahl der Probanden

## Einschlusskriterien

- schriftliche Einwilligungserklärung
- Männer und Frauen im Alter von 18 - 60 Jahren
- 25-OH-VD Konzentration im Serum ≤50 nmol/l (≤20 ng/ml)*[[2]](#footnote-3)*
- bei Frauen im gebärfähigen Alter: Anwendung einer effektiven Kontrazeption (siehe Kapitel 7.7.6)

## Ausschlusskriterien

- 25-OH-VD Konzentration im Serum >50 nmol/l
- BMI <18,5 oder >30 kg/m²
- Regelmäßige oder geplante UV-Exposition, z.B. Sonnenstudio >1x pro Woche, Reise an einen anderen Ort mit verstärkter UV-Exposition (UV-Index/Tag >5)
- Überempfindlichkeit gegen einen der Bestandteile von D3-Vicotrat® oder Vergleichspräparat
- Anamnestisch: Hyperkalzämie/-urie, Neigung zu Nierensteine, Niereninsuffizienz, Sarkoidose, Pseudohypoparathyreodismus
- Personen mit hereditärer Fructose-Intoleranz
- Einnahme von Glukokortikoiden
- Behandlung mit Vitamin A-Derivaten oder anderen Vitamin D-haltigen Präparaten
- Behandlung mit immunsuppressiv und/oder immunmodulatorisch wirksamen Medikamenten
- Behandlung mit Phenytoin, Barbiturate, Thiazid-Diuretika, Herzglykoside
- immobile Personen
- Kalzium, Phosphat oder Kreatinin im Serum mit Werten außerhalb des entsprechenden Referenzbereiches (Anlage 6), klinisch relevante Abweichungen der hämatologischen Werte (Differenzialblutbild)
- Unterbringung in einer Anstalt auf gerichtliche oder behördliche Anordnung
- (geplante) Schwangerschaft und Stillzeit
- bestehende Abhängigkeit zum Sponsor, bevollmächtigten Vertreterin des Sponsors, z.B. Verwandtschaft, Arbeitsverhältnis)
- gleichzeitige Teilnahme an einer anderen Interventionsstudie innerhalb 30 Tage vor und während der hier geplanten Studie
- sonstige Gründe, wie Drogen- und Alkoholabusus, zu erwartende fehlende Compliance

## Nicht erlaubte Medikamente

- Innerhalb 4 Wochen vor Beginn sowie während des gesamten Zeitraums der klinischen wissenschaftlichen Untersuchung
  - systemische Glukokortikoide
  - Immunsuppressiva
  - Immunmodulatoren
  - Vitamin A Derivate oder anderen Vitamin D-haltigen Präparaten
  - Phenytoin, Barbiturate, Thiazid-Diuretika, Herzglykoside

# Behandlungsplan

## Studienmedikation

Die Bezeichnung Studienmedikation bezieht sich immer auf Verum (Vitamin D3) und Placebo (NaCl 0,9%). Die Studienmedikation darf nur für diese Untersuchung verwendet werden.

### Allgemeine Information zu D3-Vicotrat®

Die Studienmedikation wird über die Firma HEYL bezogen und durch die Charité Apotheke etikettiert.

Die Studienmedikation wird unverblindet bereitgestellt und als klinisches Prüfpräparat gekennzeichnet. Der doppel-blind Charakter bleibt erhalten, indem eine nicht verblindete, studienunabhängige Person die Applikation übernimmt.

### Beschreibung der Studienmedikation

Verum (Vitamin D3)

Bezeichnung: D3-Vicotrat®

Zulassungsinhaber: HEYL Chem.-pharm. Fabrik GmbH & Co. KG, Goerzallee 253, 14167 Berlin; Zulassungsnummer: 6813051.00.00

Wirkstoff: Cholecalciferol (Vitamin D3)
1 Ampulle mit 1 ml Injektionslösung enthält 2,5 mg Cholecalciferol, entsprechend 100.000 I.E. Vitamin D3.

Sonstige Bestandteile: Natriumdihydrogenphosphat-Dihydrat, Natriumhydroxid, Sorbitollösung 70 % (kristallisierend), Polysorbat 80, mittelkettige Triglyceride, Wasser für Injektionszwecke

Haltbarkeit: Fertigarzneimittel: 3 Jahre,

Aufbewahrungshinweis: Nicht über 25°C lagern. Nach Anbruch einer Ampulle Rest verwerfen. Für Kinder unzugänglich aufbewahren.

Vergleichspräparat (Placebo)

Bezeichnung: Isotonische Natriumchloridlösung (NaCl) 0,9% Braun Injektionslösung

Hersteller: B. Braun Melsungen AG, Carl-Braun-Straße 1, 34212 Melsungen; Zulassungsnummer: 6697366.00.00

Wirkstoff: entfällt

Inhaltsstoff: NaCl 0,9 g in 100 ml Lösung

Andere Bestandteile: Wasser für Injektionszwecke

Haltbarkeit: 3 Jahre

Aufbewahrungshinweis: keine besonderen Lagerungsbedingungen erforderlich

- - 1. Äußere Form der Studienmedikation

Verum (Vitamin D3)

Darreichungsform: Glasampullen

Form und Farbe: farblose, opaleszierende Flüssigkeit

Behältnisse und Inhalt: Packungen mit 5 Ampullen á 1 ml

Vergleichspräparat (Placebo)

Darreichungsform: Kunststoffampulle aus LPDE

Form und Farbe: farblose, klare Flüssigkeit

Behältnisse und Inhalt: Kartons mit 20 Ampullen á 10 ml

- - 1. Etikettierung

Bei der Studienmedikation wird die Original-Etikettierung belassen, begründet durch die unter (14) der RICHTLINIE 2001/20/EG genannte Regelung[[3]](#footnote-4). Ein zusätzliches Etikett (Anhang 3), das die Studienmedikation als Medikation zur Anwendung im Rahmen einer klinischen Studie kennzeichnet, entsprechend der Eudralex Vol. 4 Annex 13[[4]](#footnote-5), wird durch die Charité Apotheke angebracht.

## Behandlungsschema

### Dosierung und Anwendung

Publizierte Daten zeigen, dass eine Dosis von 1.000 I.E./Tag den Vitamin D3 Status nicht substanziell erhöht . Für das sichere Erreichen einer 25-OH-VD Konzentration >50 nmol/l im Serum, als Maß einer vollen Funktionsfähigkeit von Vitamin D3 (Vitamin D3 Suffizienz) werden Vitamin D3 Mengen von 2.000 – 4.000 I.E./Tag angenommen ^2,23,41^.

In Fallberichten wurde die Sicherheit von i.m. wie auch s.c. Gabe von 100.000 I. E. Vitamin D3 gezeigt (s. Tabelle 6 im Anhang 7 aus Vieth et al. 1999). Vorteile der i.m. oder s.c. Gabe sind, dass die Dosis unabhängig von der enteralen Resorption in definierter Dosis schnell dem Körper zur Verfügung steht. Perspektivisch wäre somit eine gezielte Vitamin D3-vermittelte Immunmodulation möglich.

Die Studienmedikation wird einmalig als Einzeldosis (1 Ampulle = 1 ml = 100.000 I.E.) i.m. oder s.c. appliziert.

Die Injektionen werden durch eine nicht verblindete, studienunabhängige Person durchgeführt.

### Bereitstellung der Studienmedikation und Zuordnung zu den Behandlungs­gruppen

D3-Vicotrat® wird von Fa. HEYL zur Verfügung gestellt und über die Charité Apotheke bezogen (siehe Kap. 6.1.1). Das Vergleichspräparat wird über die Charité Apotheke bezogen.

Die Auslieferung und abschließende Rücknahme wird dokumentiert und im *Investigator’s Site File (ISF)* abgelegt, Reste der Studienmedikation werden über die Charité Apotheke entsorgt.

Sollten Mängel an der Studienmedikation festgestellt werden, erfolgt über die Fa. HEYL (Vitamin D3) bzw. die Charité Apotheke (Placebo) die Beschaffung von Ersatz.

- - 1. Lagerung, Aus- und Rückgabe

Die Studienmedikation wird bei Raumtemperatur (nicht über 25°C) temperaturkontrolliert im Studienbereich des Allergie-Centrums-Charité gelagert und die Temperatur auf dem *Temperatur Log* dokumentiert.

Die Anwendung der Studienmedikation wird für jeden Probanden auf dem entsprechenden *Drug Accountability Log* dokumentiert (siehe Kap. 11.3).

- - 1. Verzeichnis der Nebenwirkungen und Wechselwirkungen

Neben- und Wechselwirkung D_3_-Vicotrat®:

Quelle: Fachinformation (August 2009)

In den hier angewandten Dosierungen, die der zugelassenen Dosierung zur Behandlung von Vitamin D3 Mangelzuständen bei Erwachsenen entspricht, sind in dem Behandlungszeitraum keine Vitamin D3-spezifischen Nebenwirkungen zu erwarten. Nebenwirkungen entstehen nur als Folge einer Überdosierung (**Hyperkalzämie**). Bei einer akuten Symptomatik treten Herzrhythmusstörungen, Übelkeit, Erbrechen, psychische Symptome, Bewusstseinsstörungen auf. Eine chronische Symptomatik ist gekennzeichnet durch vermehrten Harndrang, verstärktes Durstgefühl, Appetitlosigkeit, Gewichtsverlust, Nierensteinbildung, Nierenverkalkung, Verkalkung in Geweben außerhalb des Knochens. In Einzelfällen sind tödliche Verläufe (bei Überdosierung) beschrieben worden. Die genannten Nebenwirkungen bilden sich nach Normalisierung der Kalziumwerte zurück.

Phenytoin oder Barbiturate sowie die gleichzeitige Verabreichung von Glukokortikoiden können die Wirkung von Vitamin D3 beeinträchtigen. Thiazid-Diuretika können durch die Verringerung der renalen Kalziumausscheidung zu einer Hyperkalzämie führen. Die Toxizität von Herzglykosiden kann infolge einer Erhöhung der Kalziumspiegel während der Behandlung mit Vitamin D3 zunehmen (Risiko für Herzrhythmusstörungen). Alle genannten wechselwirkenden Medikamente fallen unter die Ausschlusskriterien (Kap. 5.2).

Alle Risikofaktoren für Gegenanzeigen sind in den Ausschlusskriterien (Kap. 5.2) enthalten. Dazu zählen: Überempfindlichkeit gegenüber Cholecalciferol oder einem Bestandteil der Präparation, Hyperkalzämie/-urie, Neigung zu Nierensteinen, Niereninsuffizienz, Sarkoidose, Pseudohypoparathyreoidismus.

Bei i.m. Injektionen können Hämatome, Nervenschädigungen und Schmerzen insbesondere bei Belastung des jeweiligen Muskels auftreten. Bei einem Injektionsvolumen von 1 ml erachten wir diese Nebenwirkung als unwahrscheinlich vorkommend und ggf. Druckschmerz als tolerabel. Bei versehentlicher intravenöser Verabreichung kann es, durch den Ölanteil der Lösung zu Embolien und durch den Lösungsvermittler zur Hämolyse kommen (Anhang 2: klinisches Gutachten D3-Vicotrat® Fa. HEYL 2008).

Bei s.c. Injektionen können vorübergehend Hautrötung, Schmerzen und Veränderungen des Fettgewebes auftreten. Es liegen unserem Wissen nach keine Berichte über Lipdystrophien (Erkrankungen des Fettgewebes) durch Vitamin D3 vor. Ferner ist eine Granulombildung in seltenen Fällen denkbar. Aufgrund der D3-Vicotrat®-Präparation rechnen wir allerdings nicht mit spezifischen, persistierenden Granulomen, da es sich um organische Verbindungen handelt, die der Körper vollständig abbauen kann. Daher gehen wir davon aus, dass das Risiko für lokale Nebenwirkungen gering ist.

Neben- und Wechselwirkung Placebo:

Nebenwirkungen sind bei bestimmungsgemäßer Anwendung nicht zu erwarten. Wechselwirkungen sind nicht bekannt.

- - 1. Compliance

Da die Studienmedikation durch befähigtes medizinisches Personal verabreicht wird, ist eine hohe Compliance der Probanden zu erwarten.

## Begleitmedikation / Begleittherapie

Im Fall von Begleiterkrankungen und unerwünschten Ereignissen wird den Probanden auch während der Untersuchung die entsprechende Therapie erlaubt. Verbotene Medikamente sind dem Kapitel 5.3 zu entnehmen.

Alle Begleitmedikamente und Veränderungen der Begleitmedikation werden in den Originaldokumenten (*Source Data*) und im Dokumentationsbogen (*Case Report Form* - CRF) dokumentiert. Dabei werden folgende Angaben dokumentiert:

- Handelsname des Medikamentes
- Startdatum
- Enddatum
- Applikationsweg
- Dosis
- Grund für die Anwendung

## Notfallmaßnahmen

Die einzige bekannte Nebenwirkung von Vitamin D3 ist die Hyperkalzämie. Alle Risikofaktoren für Gegenanzeigen sind in den Ausschlusskriterien enthalten (siehe Kap. 5.2).

In den hier verwandten Dosierungen ist nicht mit Vitamin D3 Überdosierung zu rechnen. Bei Erwachsenen liegt die Schwelle für Intoxikationen bei 40.000 – 100.000 I.E. pro Tag über 1-2 Monate. Die in dieser klinisch wissenschaftlichen Untersuchung verabreichte Menge von einmalig 100.000 I.E. liegt weit unterhalb der Schwelle einer Intoxikation. Als zusätzliche Sicherheit werden Sicherheitslaborparameter, wie Serum-Kalzium, -Phosphat und -Kreatinin vor der Applikation der Studienmedikation sowie im Verlauf von 3 Monaten bestimmt (siehe Kap. 2). Bei den erwarteten Konzentrationen von 25-OH-VD (<200 nmol/l) sind keine Nebenwirkungen beschrieben, die Toxizitätsschwelle liegt zwischen 250-600 nmol/l ^22^.

Bei Diagnose einer Vitamin D3 Überdosierung wird neben einer kalziumarmen Diät eine stadiengerechte i.v. Gabe von isotonischer NaCl-Lsg. (3-6 L/ Tag) mit Zusatz von Furosemid sowie u.U. auch 15 mg/kg KG/Stunde Natriumedetat unter EKG/Kalzium-Kontrolle eingeleitet. Bei Oligoanurie ist eine Hämodialysetherapie indiziert.

## Randomisierung / Dekodierung

### Randomisierungsumschlag

Das Prüfzentrum von Prof. Dr. med. M. Worm (LKP) hat für jeden einzelnen Probanden einen verschlossenen Umschlag (abgeheftet im ISF), der mit der Random-Nummer beschriftet ist und die Bezeichnung der Behandlung Vitamin D3 oder Placebo sowie den Applikationsweg i.m. oder s.c. enthält.

Dieses Kuvert wird an die nicht verblindete, studienunabhängige Person, welche die Applikation durchführt am Tag der Randomisierung übergeben. Die nicht verblindete Person muss auf dem individuellen Randomisierungsumschlag das Datum der Öffnung, ihren/seinen Namen in Druckbuchstaben und ihre/seine Unterschrift dokumentieren.

Im unverblindeten ISF wird auf dem probandenspezifischen *Administration Log* die entsprechende Applikation notiert und der geöffnete Randomisierungsumschlag probandenspezifisch abgelegt.

Der unverblindeten ISF wird bis zum Ende der Studie und Datenbankschließung getrennt vom verblindeten ISF aufbewahrt.

### Notfallumschlag

Das Prüfzentrum von Prof. Dr. med. M. Worm (LKP) hat für jeden einzelnen Probanden einen zweiten verschlossenen Umschlag (Notfallkuvert, abgeheftet im verblindeten ISF). Eine vorzeitige Entblindung im Notfall erfolgt durch Öffnen dieses Notfallbriefs. Eine vorzeitige Entblindung muss im CRF und unverblindeten ISF dokumentiert werden. Dabei muss das Datum der Entblindung sowie die Unterschrift und eine kurze Begründung für die Entblindung festgehalten werden. Der LKP Prof. Dr. med. M. Worm ist unverzüglich zu informieren, wenn dieser Fall eingetreten ist. Im Fall eines SUSAR (Suspected Unexpected Serious Adverse Reaction) muss die Information an die zuständige Ethik-Kommission des Landes Berlin und die zuständige Bundesoberbehörde (BfArM) weiter geleitet werden (siehe Kap.10.5).

### Reguläre Dekodierung nach Abschluss der klinisch wissenschaftlichen Untersuchung

Die Randomisierungsliste zur Entblindung nach Studienabschluss wird in der Charité Apotheke aufbewahrt. Die reguläre Entblindung erfolgt nach Abschluss der klinisch wissenschaftlichen Untersuchung, der Datenverarbeitung, Schließung und Freigabe der Datenbank. Der Sponsor, bevollmächtigte Vertreterin des Sponsors (gleichzeitig LKP) Prof. Dr. med. M. Worm erteilt schriftlich den Auftrag zur Entblindung. Es ist zu dokumentieren, wer und wann die Entblindung vornimmt.

## Randomisierung

Die Randomisierungslisten werden von der Charité Apotheke generiert (nach Geschlechtstratifizierte Blockrandomisierung mit Sechserblöcken). Nach Evaluation der Ein- und Ausschlusskriterien werden die Probanden randomisiert. Die Zuweisung der Random-Nummern an den einzelnen Probanden erfolgt in aufsteigender Reihenfolge zur Visite 1.

# Studienablauf

Zur Veranschaulichung des Untersuchungsablaufes siehe Abb. 7-1 und Ablaufschema Kapitel 2.

Screen

Nov./Dez.
2012

| T1 T2 T3 T4  0 +2 6 24 48 72h  x x x x x x | T7  x | T14  x | T21  x | T28  x | T35  x | T42  x | T49  x | T56  x | T84  x |
| --- | --- | --- | --- | --- | --- | --- | --- | --- | --- |

4 Wochen

4 Wochen

4 Wochen

**Abb. 7-1: Graphische Darstellung des Studienablaufes;** x = Pharmakokinetik, ↓ Vitamin D3 / Placebo Applikation (i.m. oder s.c.),  = immunologische Untersuchungen

## Verfahren der Rekrutierung / des Screenings

Interessierte Probanden werden über das Prüfzentrum des Allergie-Centrum-Charité rekrutiert. Ein Anzeigetext zur Unterstützung der Probandenrekrutierung über Internet und Aushang ist dem **Anhang 4** zu entnehmen.

## Verfahren der Einwilligung nach Aufklärung

Die Probanden werden durch den behandelnden Prüfarzt ausführlich über Ziel, Dauer, Ablauf, Nutzen, Risiken und Nebenwirkungen der klinisch wissenschaftlichen Untersuchung mündlich und schriftlich aufgeklärt. Die Probanden erhalten genügend Zeit zum Lesen und Verstehen der Probandenaufklärung. In einem ausführlichen Aufklärungsgespräch mit dem behandelnden Prüfarzt hat der Proband die Möglichkeit alle bestehenden Fragen zu stellen. Die schriftliche Einwilligung wird nach ausreichender Bedenkzeit eingeholt. Erst nach dem Vorliegen der schriftlichen Einwilligung erfolgen die entsprechenden Untersuchungen.

## Verfahren zur Vermeidung von zeitgleichem Einschluss in mehreren Studien

Durch die Ausschlusskriterien wird festgelegt, dass eine gleichzeitige Teilnahme an weiteren klinischen Interventionsstudien oder Teilnahme innerhalb der letzten 30 Tage vor der klinisch wissenschaftlichen Untersuchung nicht gestattet ist. Es ist die Pflicht des Probanden das Prüfzentrum über dementsprechende Aktivitäten zu informieren. In der Probandeninformation ist unter Abschnitt „Pflichten das Probanden“ schriftlich erwähnt, dass der Teilnehmer nicht gleichzeitig an mehreren Interventionsstudien teilnehmen darf.

## Aufnahme und Registrierung

Probanden, die schriftlich und mündlich über die klinisch wissenschaftliche Untersuchung aufgeklärt wurden und ihre schriftliche Einwilligung zur Teilnahme an der Untersuchung gegeben haben, werden in einer Eingangsuntersuchung (Screening) studienspezifisch untersucht. Alle Probanden mit schriftlicher Einwilligung erhalten eine Screening-Nummer und werden in der Probandenidentifikationsliste registriert (Kap. 13). Im *Screening/Enrolment-Log* wird die Screening-Nummer mit der Random-Nummer verbunden. Die Random-Nummer wird vgl. Kapitel 6.6 vergeben.

## Klinische Untersuchungen und Abweichungen von der üblichen klinischen Praxis

Während der klinisch wissenschaftlichen Untersuchung werden folgende Untersuchungsmethoden angewendet, die auch in der routinemäßigen Diagnostik zum Einsatz kommen: Anamnese, körperliche Untersuchung und Messung der Vitalparameter, Blutentnahmen für das Sicherheitslabor.

Zu den studienspezifischen Maßnahmen und Untersuchungen gehört die Abfrage der Ein- und Ausschlusskriterien, der Schwangerschaftstest für gebärfähige Frauen, die verblindete i.m. oder s.c. Applikation der Studienmedikation und Dokumentation dieser, Dokumentation evtl. auftretende unerwünschter Ereignisse, Blutentnahme für die Pharmakokinetik und immunologischen Parameter.

- - 1. Eingangs- / Einschlussuntersuchung

Bei der Eingangsuntersuchung werden die Ein- und Ausschlusskriterien abgefragt. Die Eignung des Probanden zur Teilnahme an der klinisch wissenschaftlichen Untersuchung wird überprüft, relevante Basisdaten werden erhoben.

Das Untersuchungsprogramm zur Screening Visite enthält:

- Anamnese, einschließlich Dokumentation aktuellen Medikamente
- Dokumentation der habituellen UV-Exposition
- körperliche Untersuchung, Vital-Parameter, Bestimmung Körpergröße und ‑gewicht
- Blutentnahme für Sicherheitslabor
- 25-OH-VD Messung
- Schwangerschaftstest bei gebärfähigen Frauen
  - 1. Visite 1 (Randomisierung und Behandlungsstart)

Visite 1 sollte nicht länger als 4 Wochen nach der Eingangsuntersuchung stattfinden. Zu Visite 1 und allen folgenden Visiten werden 10 €/Visite als Aufwandsentschädigung gewährt. Das Untersuchungsprogramm zur Visite 1 enthält:

- Kontrolle, der Ein- und Ausschlusskriterien
- Dokumentation von Begleitmedikation und unerwünschter Ereignisse (*Adverse Event* – AE)
- Randomisierung und Applikation der Studienmedikation
- Blutentnahme für immunologische Parameter (vor Applikation der Studienmedikation)
- Blutentnahmen für die Pharmakokinetik (vor, 1h, 2h, 6h)
- Dokumentation der Verträglichkeit der Studienmedikation
  - 1. Visiten 2 (1 Tag), V3 (2 Tage) und V4 (3 Tage) nach Applikation der Studienmedikation)

Das Programm dieser Visiten enthält:

- Blutentnahmen für die Pharmakokinetik
- Dokumentation von Begleitmedikation und AEs
  - 1. Visite 5 (7 ±1 Tage nach Applikation der Studienmedikation)

Das Programm der Visiten 5:

- Blutentnahme für immunologische Parameter (primärer Endpunkt), bei männlichen Probanden werden hier 200 ml anstatt 20 ml abgenommen
- Blutentnahmen für die Pharmakokinetik
- Dokumentation von Begleitmedikation und AEs
  - 1. Visite 6, 7, 8, 9 und 10

Das Programm dieser Visiten enthält:

- Blutentnahmen für die Pharmakokinetik
- Dokumentation von Begleitmedikation und AEs
  - 1. Visite 11 (4 Wochen nach Applikation der Studienmedikation)

Das Untersuchungsprogramm zur Visite 11 enthält:

- Vital-Parameter
- Blutentnahme für Sicherheitslabor und immunologische Parameter, bei männlichen Probanden werden hier 200 ml anstatt 20 ml abgenommen
- Blutentnahmen für die Pharmakokinetik
- Dokumentation von Begleitmedikation und AEs
  - 1. Visite 12 (8 Wochen nach Applikation der Studienmedikation)

Das Untersuchungsprogramm zur Visite 12 enthält:

- Vitalparameter
- Blutentnahme für Sicherheitslabor
- Blutentnahmen für die Pharmakokinetik
- Dokumentation von Begleitmedikation und AEs
  - 1. Visite 13 (12 Wochen nach Applikation der Studienmedikation, Abschlussvisite)

Das Untersuchungsprogramm zur Visite 13 enthält:

- Vitalparameter
- Blutentnahme für Sicherheitslabor und immunologische Parameter
- Blutentnahmen für die Pharmakokinetik
- Dokumentation von Begleitmedikation und AEs
  - 1. Laboruntersuchungen

Folgende Mengen Blut werden an folgenden Visiten abgenommen:

| Blutentnahmen | Screen | V1 | V2 | V3 | V4 | V5 | V6, V7, V9-11 | V8 | V12 | V13 |
| --- | --- | --- | --- | --- | --- | --- | --- | --- | --- | --- |
| Sicherheitslabor  Immunologie  Pharmakokinetik | 5 ml  10 ml* | 20 ml  4x 10 ml | 10 ml | 10 ml | 10 ml | 20ml  10 ml | 10 ml | 5 ml  20/200^**^ ml  10 ml | 5 ml  10 ml | 5 ml  20 ml  10 ml |
| Gesamt-Menge | 15 ml | 60 ml | 10 ml | 10 ml | 10 ml | 30/210^**^ ml | 10 ml | 35/215^**^ ml | 15 ml | 35 ml |

*Hier erfolgt nur die Messung von 25-OH-VD

**bei männlichen Probanden werden hier 200 ml abgenommen

## Beurteilung der Wirksamkeit

### Immunologische Wirksamkeit

Im Rahmen dieser klinisch wissenschaftlichen Pilot-Untersuchung erfolgt eine rein explorative, deskriptive Auswertung. Ein Wirksamkeitsnachweis i.S. einer vergleichenden klinischen Studie ist nicht vorgesehen.

*Häufigkeit Vitamin D3-sensitiver Immunzellen*

Das Ziel der klinisch wissenschaftlichen Untersuchung ist die Kinetik von Immunzellen (T- und B-Lymphozyten, myeloide APZs) im Blut zu bestimmen, die durch endogene Calcitriolsynthese moduliert wurden. Wir möchten untersuchen, ob nach Vitamin D3-Gabe die Häufigkeiten von Vitamin D-sensitiven T- und B-Lymphozyten, sowie myeloiden APZ im Blut verändert sind.

Die Erfassung des Zeitpunktes, ab wann und über welchen Zeitraum diese im Blut zirkulieren ist Gegenstand dieser Untersuchung. Die Zellmessungen erfolgen vor Vitamin D3 Gabe an Tag 1, sowie an 7 Tage, 28 Tage und 84 Tage nach der i.m. bzw. s.c. Applikation.

Periphere Blutzellen werden mit fluoreszenzgekoppelten monoklonalen Antikörpern spezifisch angefärbt und anschließend werden 1.000.000 Zielzellen durchflusszytometrisch gemessen. Anschließend wird die Häufigkeit von Immunzellen analysiert, die klassische Vitamin D3-responsive Oberflächenmarker aufweisen, die als Surrogatmarker dienen; im Detail:

CD38^+^CD19^+^ B-Zellen, CD38^+^CD4^+^ T-Zellen, oder HLA-DR^low^CD14^+^ Monozyten (im Folgenden Vitamin D-sensible Immunzellen genannt).

Ferner möchten wir den Phänotyp der Vitamin D3-Immunzellen bestimmen. Dies erfolgt durch Kombination der o.g. Vitamin D3-abhängigen Marker mit spezifischen Subpopulationsmarkern auf CD4^+^ T-Zellen: CCR7 und CD45RA, sowie Aktivierungsmarker CD154^+^; auf CD19^+^B-Zellen: CD27 und IgM, sowie CD16^+^ auf Monozyten. Dies erlaubt die qualitative Aussage ob es sich um folgende Zellpopulationen handelt:

T-Zellen: naive (CD45RA+), Gedächtnis (CD45RA-), Central effector (CCR7+), Central memory (CCR7-), oder aktiviert (CD154+CD4+).

B-Zellen: naive (CD27-IgM+), transitional (CD27+IgM) oder Gedächtnis (CD27+IgM-) oder

myeloide APZ: inflammatorische (CD16+) oder ruhende Monozyten (CD16-).

Somit können qualitative Aussagen getroffen werden, welche Zellen genau Vitamin D-sensibel sind, was eine Spekulation über deren Herkunft und mögliche biologische Funktion erlaubt.

Ferner möchten wir weitere immunologische Parameter erfassen:

- Häufigkeit von Zytokin-produzierenden CD4^+^ T Helferzellen: IFN-γ^+^, IL-17^+^, IL-4^+^, IL­10^+^.
- Aktivierung von myeloider APZ: CD38 Expression auf HLA-DR^low^ CD14^+^ Monozyten.
- sezernierte anti-EBV-, anti-CMV-, anti-VZV-spezifische Immunglobulinkonzentration: mittels ELISA von Seren der Klassen IgG1, IgG2, IgA).

Bei männlichen Probanden werden zu Visite 5 und 8 jeweils 200 ml Blut zur Bestimmung von Transkriptomen von Vitamin D3-reagierenden Zellen abgenommen. Aus dem Blut werden 200.000 Vitamin D3-reagierende B- und T-Zellen sowie definierte Kontrollpopulationen durchflusszytometrisch identifiziert, isoliert und anschließend eingefroren. Nach Entblindung der Studie werden die Proben von Probanden mit Vitamin D-Gabe (i.m. oder s.c.) weiter molekularbiologisch aufgearbeitet. Analoge Untersuchungen aus früheren *in vitro* Experimenten zeigen ca. 200 VDR-Zielgene in B-Zellen. Eine Auswahl von Zielgenen wollen wir mit denen vergleichen, die *in vivo* durch die Vitamin D3 Applikation reguliert sind. Diese translationalen Untersuchungen ergänzen unsere zuvor experimentell gewonnenen Daten und helfen das molekulare Verständnis zur immunologischen Wirkung von Vitamin D3 zu verbessern. Perspektivisch werden die *in vivo* und *in vitro* identifizierten Gene funktionell untersucht.

**Pharmakokinetik**

Die Messung der Vitamin D3 Metaboliten erfolgt vor Applikation der Studienmedikation, nach 1 h, 2 h, 6 h, 24 h , 48 h, 72 h, sowie wöchentlich von Woche 1-9 (V1-13).

Für die Pharmakokinetik wird der Vitamin D3 Speichermetabolit 25-Hydroxyvitamin D (zeigt Provitamin D Verfügbarkeit) im zertifizierten Zentrallabor der Charité - Universitätsmedizin Berlin, Institut für Laboratoriumsmedizin, Klinische Chemie und Pathobiochemie (Anhang 5: Referenzbereiche) gemessen.

Explorativ werden Vitamin D3 selbst (zeigt Anflutung) und folgende Vitamin D3 Metabolite im Serum gemessen: Calcitriol (biologisch aktive Metabolit, chem. 1,25-Dihydroxyvitamin D) und 1,24,25-Calcitroinsäure (zeigt Abbauprodukt). Das benötigte Material wird für diese Messungen eingefroren und gesammelt und zu späterem Zeitpunkt gemessen. Durch den Wegzug unseres Kooperationspartners Prof. Dr. T. Unger, Charité Campus Mitte, Institut für Pharmakologie wird ein neuer Kooperationspartner gesucht und entsprechend durch den Sponsor beauftragt. Unverändert werden die Messungen der Pharmakokinetik extern erfolgen und die Daten werden von einer studienunabhängigen Person kontrolliert, um den doppel-blind Charakter zu bewahren. Bei medizinisch auffälligen Befunden wird der Studienarzt kontaktiert, wobei die Daten pseudonymisiert behandelt werden.

## Sicherheit und Verträglichkeit

### Anamnese

Mit der Anamnese wird sichergestellt, dass Probanden nicht an interferierenden Grunderkrankungen leiden. Zudem werden alle dauerhaften Begleitmedikamente aufgenommen.

Ferner wird die habituelle oder geplante UV-Exposition eruiert.

### Körperliche Untersuchung und Vitalparameter

Bei allen Probanden wird vor der Behandlung eine körperliche Untersuchung, inkl. Vitalparameter durchgeführt. Zu den Vitalparametern zählen Blutdruck und Puls. Diese werden auch während und nach der Behandlung kontrolliert (**Anhang 5**: Referenzbereiche).

### Unerwünschte Ereignisse

Unerwünschte Ereignisse (AE) werden durch das Studienpersonal bei jeder Visite erfasst. Die aufgetretenen AEs werden in den dafür vorgesehenen CRFs dokumentiert (siehe Kap. 10.4).

### Sicherheitslabor

Die Bestimmung der Sicherheitslaborparameter erfolgt im zertifizierten Zentrallabor der Charité - Universitätsmedizin Berlin, Institut für Laboratoriumsmedizin, Klinische Chemie und Pathobiochemie (**Anhang 5**: Referenzbereiche). Die Bestimmung erfolgt vor der Randomisierung (Screening), zur Visite 5 (4 Wochen nach der Applikation der Studienmedikation), zur Visite 9 (8 Wochen nach der Applikation der Studienmedikation) und zur Visite 10 (12 Wochen nach der Applikation der Studienmedikation), sowie bei Bedarf. Klinisch relevante Abweichungen sind als Ausschlusskriterium definiert.

Mit der Bestimmung der Sicherheitslaborparameter vor Beginn, während und am Ende der klinisch wissenschaftlichen Untersuchung soll einerseits sichergestellt werden, dass keine Probanden mit interferierenden Grunderkrankungen eingeschlossen werden und andererseits die biochemische Verträglichkeit dokumentiert wird. Eine deskriptive Auswertung der Sicherheitsparameter wird der Publikation zugefügt.

### Verträglichkeit

Die Verträglichkeit wird 2, 6, 24, 48 und 72 h nach Applikation der Studienmedikation durch den Probanden selbst und den Prüfarzt nach folgender Skala beurteilt:

1. sehr gut

2. gut

3. mäßig

4. schlecht

5. sehr schlecht

### Schwangerschaftstest und Kontrazeption

Bei Frauen wird vor Beginn der Vitamin D3 Applikation ein Urin-Schwangerschaftstest durchgeführt. Ferner wird der Test während der Behandlung durchgeführt, wenn die Probandinnen einen Verdacht auf Schwangerschaft äußern (Menstruation bleibt aus).

Frauen im gebärfähigen Alter müssen bei Einschluss und während der gesamten klinisch wissenschaftlichen Untersuchung eine effektive Methode zur Kontrazeption (Versagerquote < 1%) anwenden. Dazu zählen u.a. Sterilisation, orale hormonelle Kontrazeption (Pille), 3-Monats-Spritze, Hormonimplantat, Hormonspirale, Vaginalring, Transdermalpflaster und/oder und ein sterilisierter Partner). Nicht zur Verhütung geeignet sind folgende Methoden: die einfache Anwendung von Kondom, Diaphragma oder Spermizide, die Temperaturmethode, sexuelle Abstinenz oder Minipille.

## Dauer der Teilnahme an der klinisch wissenschaftlichen Untersuchung für den einzelnen Probanden

Bei regulärer Durchführung und Beendigung der klinisch wissenschaftlichen Untersuchung erhält jeder Teilnehmer eine Applikation der Studienmedikation (i.m. oder s.c.) und wird über insgesamt 12 Wochen klinisch kontrolliert (siehe Abb. 7-1).

# Risiko-Nutzen-Abwägung

D_3_-Vicotrat® ist seit fast 40 Jahren im Handel und wurde in der Bundesrepublik Deutschland unter der Nr. D 1194 am 06. Dezember 1968 beim Bundesgesundheitsamt registriert. Das Präparat wurde nach Umstellung der rechtlichen Vorschriften (AMG 1976) in Deutschland am 02. Februar 1999 auf Basis der eingereichten Unterlagen unter der Zulassungsnummer 6813051.00.00 zugelassen. Die erste Verlängerung der Zulassung nach § 31 AMG wurde mit Bescheid vom 23. November 2005 bestätigt. D_3_-Vicotrat® ist das einzige, in Deutschland zugelassene Vitamin-D_3_-Präparat zur i.m. Applikation.

Zu den Nebenwirkungen zählen die Hyperkalzämie-bedingte Phänomene. Abhängig von Dosis und Behandlungsdauer kann eine schwere und lang anhaltende Hyperkalzämie mit ihren akuten Folgen (Herzrhythmusstörungen, Übelkeit, Erbrechen, psychische Symptome, Bewusstseinsstörungen) und chronischen Folgen (Polyurie, Polydipsie, Inappetenz, Gewichtsverlust, Nierensteinbildung, Nephrocalcinose, extraossäre Verkalkungen) auftreten. Besondere Vorsicht ist geboten bei Personen mit organischer Prädisposition (verstärkte/unkontrollierte Calcitriolsynthese/ Sarkoidose oder verminderte Ausscheidung/ Niereninsuffizienz).

Es existieren keine Berichte über Vitamin D3-vermittelte Nebenwirkungen der im Rahmen dieser Studie einmaligen Gabe von 100.000 I.E. (Stand Mai 2012). Über nachteilige immunmodulatorische Wirkungen von Vitamin D3 in den hier erwarteten Serumkonzentrationen (115 ±7,1 nmol/l) liegen keine Berichte vor. Im Gegenteil wird mit den hier verwandten Vitamin D3 Mengen der Vitamin D3 Mangel (Einschlusskriterium) ausgeglichen, was positiv ist und für den Knochenstoffwechsel empfohlen wird und auch für günstige immunologische Funktionen diskutiert wird.

Das Risiko einer Hyperkalzämie durch die Vitamin D3 Applikation ist als sehr gering einzustufen, da alle Probanden zu Beginn einen Vitamin D3 Mangel aufweisen. Durch zahlreiche Untersuchungen wurde gezeigt, dass die vorgesehene Dosierung als sicher einzustufen ist (siehe Kap. 6.2.1 und 6.2.4.).

Durch die Einmaldosis von 100.000 I.E. mittels i.m. bzw. s.c. Injektion können definierte Zielkonzentrationen von Provitamin D (25-OH-VD) erzielt werden, da keine Abhängigkeit zur Resorptionsrate im Darm besteht. Zudem kann im Gegensatz zur täglichen Einnahme eine 100%-ige Compliance erzielt werden und der Vitamin D3 Mangel schnell ausgeglichen werden.

Somit können auch Vitamin D3-reagierende Immunzellen im Blut günstig nachgewiesen werden, da sie gleichzeitig auf Vitamin D3 reagieren und nicht sequenziell, wie es bei einer täglichen oralen Einnahme der Fall wäre.

Vitamin D-Mangel ist in der Bevölkerung häufig in den Wintermonaten (über 50% bei Erwachsenen in Deutschland) ^3^. Insofern ist ein unmittelbarer körperlicher Schaden wenig wahrscheinlich, langfristig ist ein erhöhtes Risiko für Osteopenie /-porose bekannt. Im Sommer reichen ca. 15 Min. tägliche UV-Exposition für ausreichende Vitamin D Synthese aus. Entsprechend werden alle Probanden über Massnahmen zur Prophylaxe, z.B. durch gemäßigte Sonnenexposition im Sommer sowie, nach entsprechender Kontrolle der 25-Hydroxyvitamin D Serumwerte, Vitamin D Supplementation in den Wintermonaten, aufgeklärt.

Die Blutentnahmen in der vorliegenden klinisch wissenschaftlichen Untersuchung stellen kein untersuchungsspezifisches zusätzliches Risiko dar. Zu zwei Messzeitpunkten (Visite 2 und 5) wird eine größere Menge Blut (200 ml) entnommen. Dieses Volumen entspricht etwas mehr als 1/3 von dem, was bei einer Blutspende entnommen wird. Aufgrund des damit verbundenen Eisenverlustes werden diese zusätzlichen Blutentnahmen nur bei männlichen Probanden abgenommen.

# Abbruch und Weiterbehandlung

## Vorzeitiger Studienabbruch eines einzelnen Probanden

Wenn bei einem Probanden eines oder mehrere der folgenden Ereignisse auftreten, wird die klinisch wissenschaftliche Untersuchung in Absprache mit dem Prüfarzt und dem LKP für diesen Probanden abgebrochen:

- persönlicher Wunsch des Probanden
- Eintritt einer Schwangerschaft
- nicht ausreichende Compliance
- Einnahme oder Anwendung von Medikamente, die ein Ausschlusskriterium darstellen (Kap. 5.2)
- Hyperkalzämie
- Jede Situation, die nach Ansicht des Prüfarztes einer weiteren Teilnahme an der klinisch wissenschaftlichen Untersuchung des Probanden entgegen sprechen würde.

Im Fall des Auftretens einer Hyperkalzämie wird durch den Prüfarzt eine angemessene Therapie eingeleitet und solange kontrolliert bis sich der Gesundheitszustand des Probanden auf den Ausgangspunkt vor Beginn der Untersuchung erholt hat.

## Vorzeitiger Abbruch der gesamten klinischen Prüfung

Der Sponsor, bevollmächtigte Vertreterin des Sponsors (gleichzeitig LKP) Prof. Dr. med. M. Worm hat das Recht, die Untersuchung vorzeitig zu beenden:

- bei unvertretbaren Risiken und Toxizitäten unter Nutzen-Risiko-Abwägung
- beim Auftreten neuer Erkenntnisse während der Laufzeit der klinisch wissenschaftlichen Untersuchung, die die Sicherheit der Studienteilnehmer gefährden können (positive Nutzen-Risiko-Abwägung nicht mehr gegeben)

Zudem liegt es im Ermessen der Ethik-Kommission des Landes Berlin die klinisch wissenschaftliche Untersuchung jederzeit zu beenden.

## Plan für die Weiterbehandlung/medizinische Betreuung

Bei allen Probanden, die aus der klinisch wissenschaftlichen Untersuchung vorzeitig ausscheiden müssen, sollte eine Abschlussuntersuchung (entsprechend der Visite 10, siehe Kap. 7.5.8) durchgeführt und die Abschlussseite im CRF ausgefüllt werden.

Probanden die aufgrund unerwünschter Ereignisse ausscheiden, die unmittelbar mit dem zu prüfenden Arzneimittel in Zusammenhang gebracht wurden, werden solange nach beobachtet, bis sich die Befunde verbessert haben.

# Unerwünschte Ereignisse

Im folgenden Kapitel ist mit Sponsor immer die bevollmächtigte Vertreterin des Sponsors (gleichzeitig LKP) Prof. Dr. med. M. Worm gemeint.

Jegliche Informationen über unerwünschte Ereignisse (*Adverse Event* - AE), entweder freiwillig durch den Probanden genannt oder durch den Prüfarzt erfragt, werden dokumentiert. Informationen über schwerwiegende unerwünschte Ereignisse (*Serious Adverse Event* - SAE) werden unverzüglich (jedoch innerhalb 24 h) dem Sponsor gemeldet.

Für die Abfrage, Beurteilung und Dokumentation von AEs und SAEs gilt immer auch die aktuelle Verfahrensanweisung im Prüfzentrum des Allergie-Centrums-Charité.

## Definitionen (nach Richtlinie 2001/20/EG)

**Unerwünschtes Ereignis (Adverse Event - AE)**

„Jedes schädliche Vorkommnis das einem einem Prüfungsteilnehmer widerfährt, dem ein Arzneimittel verabreicht wurde, und das nicht unbedingt in kausalem Zusammenhang mit dieser Behandlung steht“.

Dies können Erkrankungen, Krankheitszeichen oder Symptome sein, die nach Einschluss des Probanden in die Studie eintreten oder sich verschlechtern.

Die Ausprägung wird wie folgt bewertet:

- gering
- mäßig
- schwer
- Kriterien für ein Schwerwiegendes unerwünschtes Ereignis (SAE) sind erfüllt → siehe SAE

Für jedes Ereignis ist eine Kausalitätsbewertung vorzunehmen:

- kein Zusammenhang
- möglicher Zusammenhang
- wahrscheinlicher Zusammenhang
- sicherer Zusammenhang
- nicht beurteilbar

**Schwerwiegendes unerwünschtes Ereignis (*Serious Adverse Event* - SAE)**

Schwerwiegend ist ein Unerwünschtes Ereignis, das

- tödlich oder lebensbedrohend
- eine stationäre Behandlung oder deren Verlängerung erforderlich macht
- zu einer bleibenden oder schwerwiegenden Behinderung oder Invalidität führt
- oder eine kongenitale Anomalie oder einen Geburtsfehler zur Folge hat

**„Verdachtsfall einer unerwarteten schwerwiegenden Nebenwirkung“ (Suspected Unexpected Serious Adverse Reaction - SUSAR)**

Ein Verdachtsfall einer unerwarteten schwerwiegenden Nebenwirkung (*Suspected Unexpected Serious Adverse Reaction* – SUSAR) liegt vor, wenn schädliche und unbeabsichtigte Begleiterscheinungen mit der Gabe des Arzneimittels in zeitlichem Zusammenhang stehen (auf Spätschäden muss hierbei gesondert geachtet werden) und nach angemessener Recherche andere Ursachen als die Gabe des Arzneimittels für die Begleiterscheinungen ausgeschlossen werden können.

## Beurteilung der Intensität

Leicht: Das unerwünschte Ereignis ist vorübergehend und vom Probanden leicht zu ertragen.

Mäßig: Das unerwünschte Ereignis bereitet dem Probanden Unannehmlichkeiten und behindert ihn bei seinen üblichen Tätigkeiten.

Schwer: Das unerwünschte Ereignis bereitet dem Probanden erhebliche Störungen seiner üblichen Aktivitäten.

## Beurteilung des Kausalzusammenhanges

Der Prüfarzt wird gefragt, ob ein Zusammenhang zwischen dem AE und der Studienmedikation (Vitamin D3/Placebo) besteht. Für die Beurteilung des Zusammenhangs zwischen der Anwendung der Studienmedikation und einem AE werden folgende Definitionen verwendet:

Sicher: Eine Reaktion, die einem nachvollziehbaren zeitlichen Ablauf nach der Anwendung des Prüfproduktes folgt oder bei der die Arzneimittelkonzentration in Körpergewebe oder ‑flüssigkeit gemessen wurde, einem bekannten oder erwarteten Antwortmuster auf das verdächtigte Prüfprodukt folgt und nach Absetzen oder Dosisreduktion verschwindet und bei erneuter Exposition wieder auftritt.

Wahrscheinlich: Eine Reaktion, die einem nachvollziehbaren zeitlichen Ablauf nach der Anwendung des Prüfproduktes folgt, einem bekannten oder erwarteten Antwortmuster auf das verdächtigte Prüfprodukt folgt und nach Absetzen oder Dosisreduktion verschwindet und nicht durch die bekannten Merkmale des klinischen Zustandes des Probanden erklärt werden kann.

Möglich: Eine Reaktion, die einem nachvollziehbaren zeitlichen Ablauf nach der Anwendung des Prüfproduktes folgt, einem bekannten oder erwarteten Antwortmuster auf das verdächtigte Prüfprodukt folgt, die aber leicht auch durch eine Reihe anderer Faktoren hervorgerufen worden sein könnte.

Kein Zusammenhang: Eine Reaktion, bei der ausreichend Informationen vorliegen für die Annahme, dass kein Zusammenhang mit dem Prüfprodukt besteht.

Nicht beurteilbar: Eine Einschätzung des Zusammenhangs ist nicht möglich.

## Dokumentation von AEs und SAEs

Sämtliche AEs und SAEs sind zu dokumentieren, unabhängig davon, ob nach Meinung des Prüfarztes ein ursächlicher Zusammenhang mit dem Studienmedikament besteht oder nicht. Die Dokumentation umfasst die Art des Ereignisses, Beginn, Dauer, Ende, Ausprägung/Schweregrad (Intensität) und Kausalität.

Im Zusammenhang stehende Krankheitszeichen, Symptome und Laborwertveränderungen sollen zu einer einzigen Erkrankung zusammengefasst werden. Für die Dokumentation stehen die AE-Bögen im CRF zur Verfügung. SAEs werden zusätzlich auf einem gesonderten SAE-Bogen dokumentiert.

Alle AEs und SAEs sind bis zum Abklingen oder bis zur Stabilisierung zu verfolgen.

Ausnahmenregelung

Im Rahmen dieser klinisch wissenschaftlichen Untersuchung sollen die folgenden AEs/SAEs von der Meldepflicht ausgenommen werden:

- AE/SAE, das nach Einschluss, jedoch vor Behandlungsbeginn eintritt.
- Weitere Ereignisse: z.B. vor Studieneinschluss bereits bekannte (angekündigte) Ereignisse, die mit einer Hospitalisierung einhergehen (z.B. erwartete OPs).

## Meldung von SAEs und von Verdachtsfällen schwerwiegender unerwarteter unerwünschter Nebenwirkungen (SUSARs)

Tab. 10-1: Meldeverpflichtungen und -fristen in der klinisch wissenschaftlichen Untersuchung für SAEs/SUSARs (nach GCP-V §§ 12, 13 und Leitlinie ENTR/CT 3)

**A) Prüfarzt 🡺 Sponsor**

| **Art** | **Frist** | **Bemerkung** |
| --- | --- | --- |
| SAE | Unverzüglich (spätestens aber innerhalb von 24 h) | Schriftlicher Bericht (SAE-Bogen) |

**B) Sponsor 🡺 Behörden / Prüfärzte**

| **Art** | **Frist** | **EK*** | **BOB**** | **Prüfärzte** | **Fa. HEYL** |
| --- | --- | --- | --- | --- | --- |
| SAE | Auf Anforderung |  | x |  | x |
| SUSAR | Einzelfallbericht innerhalb von 15 Tagen | x | x | x | x |
| SUSAR (Tod)    Folgemeldung | Einzelfallbericht innerhalb von 7 Tagen  Weitere 8 Tage | x  x | x  x | x  x | x  x |

* EK – zuständigen Ethikkommission, hier: Ethikkommission des Landes Berlin

** BOB – zuständigen Bundesoberbehörde, hier: Bundesinstitut für Arzneimittel und Medizinprodukte (BfArM)

Im Fall des Auftretens eines SAEs muss der Prüfarzt die entsprechenden SAE-Bögen ausfüllen. Der Prüfarzt unterrichtet den Sponsor unverzüglich (jedoch innerhalb 24 h) über das Auftreten eines SAEs (Tab. 10-1A). Folgende Angaben müssen dem Sponsor übermittelt werden: Random-Nummer, kurze Beschreibung des SAEs, Startdatum des ursprünglichen entsprechenden AEs. Zusätzlich muss der bevollmächtigten Vertreterin des Sponsors der SAE-Bogen schnellstmöglich (innerhalb von 2 Werktagen) vorliegen.

Falls der Sponsor die Notwendigkeit sieht oder auf Anforderung, sind SAEs der zuständigen Bundesoberbehörde (BfArM) und der Fa. HEYL zu melden (Tab. 10-1B).

Schwangerschaft fällt nicht unter die Definition eines AEs bzw. SAEs, muss dem Sponsor aber auch innerhalb von 24 h gemeldet werden.

Wird dem Sponsor deutlich, dass ein aufgetretenes SAE mit der Studienmedikation direkt im Zusammenhang steht (Verdachtsfall einer unerwarteten schwerwiegenden Nebenwirkung - SUSAR), muss er dies unverzüglich, spätestens aber innerhalb 15 Tage nach Bekannt werden der Ethik-Kommission des Landes Berlin und dem BfArM melden. Weiterhin muss er alle an der klinisch wissenschaftlichen Untersuchung beteiligten Prüfärzte und die Fa. HEYL darüber unterrichtet.

Todesfälle sollten innerhalb von 7 Tagen gemeldet werden und nach Möglichkeit sollte eine Kopie des Autopsieberichts beiliegen. Alle weiteren relevanten Informationen sollten innerhalb von höchsten 8 weiteren Tagen übermittelt werden.

Der Sponsor unterrichtet unverzüglich, spätestens aber innerhalb von 15 Tagen nach Bekanntwerden, die Ethik-Kommission des Landes Berlin und das BfArM über jeden Sachverhalt, der eine erneute Überprüfung der Nutzen-Risiko-Bewertung der klinisch wissenschaftlichen Untersuchung erfordert. Hierzu gehören insbesondere:

- - Einzelfallberichte von erwarteten schwerwiegenden Nebenwirkungen der Studienmedikation mit einem unerwarteten Ausgang.
  - Erhöhung der Häufigkeit erwarteter schwerwiegender Nebenwirkungen, die als klinisch relevant bewertet wird.

Personenbezogene Daten werden vor ihrer Übermittlung immer pseudonymisiert. Vor Meldung eines SUSARs wird die Blindung für diesen Probanden aufgehoben.

## Schwangerschaften

Schwangerschaft ist nicht als AE oder SAE zu klassifizieren. Auch AEs oder SAEs, die im Zusammenhang mit einer Schwangerschaft auftreten, sind nicht als solches zu dokumentieren. Das Auftreten einer Schwangerschaft muss innerhalb von 24 h an den Sponsor gemeldet werden

Vitamin D3 ist ein physiologisches Hormon des Menschen und wird in der 25-hydroxylierten Form (25-OH-VD) in Fett- und Muskelgeweben gespeichert. Auswirkungen in normo-kalzämischen Konzentrationen auf Schwangerschaft und Stillzeit sind daher nicht zu erwarten.

Überdosierungen von Vitamin D3 in der Schwangerschaft müssen verhindert werden, da eine lang anhaltende Hyperkalzämie zu körperlicher und geistiger Behinderung sowie angeborenen Herz- und Augenerkrankungen des Kindes führen kann (Fachinformation D3-Vicotrat® August 2009). D3-Vicotrat® darf deswegen in der Schwangerschaft und Stillzeit nicht angewendet werden.

Aus medizinischen wie auch ethischen Gründen wird eine schwangere Probandin sofort von der klinisch wissenschaftlichen Untersuchung ausgeschlossen.

Ist zu dem Zeitpunkt der Diagnose einer Schwangerschaft eine Hyperkalzämie nachweisbar, werden stadiengerechten Gegenmaßnahmen ergriffen, sowie im Verlauf die Entwicklung des Kindes überwacht.

# Dokumentation

## Dokumentationsbögen (CRF)

Die erhobenen Daten werden im Papier-CRF dokumentiert.

## Prüfarztordner (ISF)

Im Prüfarztordner (Investigator’s Site File – ISF) werden alle essentiellen Dokumente gemäß ICH GCP Kapitel 8 im Prüfzentrum vor Ort abgelegt.

## Dokumentation der Studienmedikation (*Drug Accountability*)

Die Applikation der Studienmedikation laut Behandlungsplan (Kap. 2) wird im probandenspezifischen *Drug Accountability Log* aufgezeichnet. Folgende Angaben werden auf dem *Drug Accountability Log* abgefragt: Random-Nummer, Datum der Applikation, Art und Menge der Applikation, die Visite, die Chargennummer, Datum und Unterschrift des Ausgebenden.

Die *Drug Accountability Logs* werden im ISF abgelegt. Neben der Source Data Dokumentation kann zusätzlich eine Kopie der *Drug Accountability Logs* erfolgen.

Die Lieferung sowie Rücksendung der Präparate von bzw. zur Charité Apotheke werden im ISF dokumentiert.

# Qualitätsmanagement

Die Planung und Durchführung der klinisch wissenschaftlichen Untersuchungen sowie die geplanten Maßnahmen zur Qualitätssicherung erfolgen gemäß dem AMG, der GCP-V sowie den geltenden gesetzlichen Bestimmungen und halten sich an die Empfehlungen der Deklaration von Helsinki und den ICH-GCP-Richtlinien.

**Überwachung des Studienablaufs und der Datenqualität - Monitoring**

Der Prüfarzt ist verantwortlich für die vollständige Dokumentation in der Krankenakte und in den CRF. Zudem ist der Prüfarzt verpflichten die Einhaltung des Protokolls zu gewährleisten. Alle *Source Data*, CRF und der ISF müssen dem klinischen Monitor und ggf. Auditoren und Inspektoren zugänglich sein.

Für die Datensicherheit, -vollständigkeit und -konsistenz wird die klinisch wissenschaftliche Untersuchung kontrolliert. Hierfür beauftragt der Sponsor, bevollmächtigte Vertreterin des Sponsors (gleichzeitig LKP) Prof. Dr. med. M. Worm eine für das klinische Monitoring befähigte Person. Der Zugang des klinischen Monitors zu den Originalakten und CRF ist gesichert. Die Häufigkeit der Monitor-Besuche und der Umfang des Monitoring werden in einem Monitor-Plan festgelegt. Bei alle Monitor-Besuchen wird ein Bericht geschrieben, der an die bevollmächtigte Vertreterin des Sponsors weitergeleitet wird.

# Dateneingabe und Datenmanagement

## Allgemeines

Alle personenbezogenen Daten werden in pseudonymisierter Form erfasst. Jeder Proband ist durch eine Screening- und Random-Nummer unverwechselbar gekennzeichnet. Die Screening-Nummer besteht aus dem Buchstaben „S“ und 2 Ziffern, beginnend mit 01 und wird in aufsteigender Reihenfolge den Probanden, die ihre Einwilligung gegeben haben, zugeordnet. Die Random-Nummer wird durch die Charité Apotheke generiert (vgl. Kap. 6.6). Auch die Random-Nummer wird in aufsteigender Reihenfolge den randomisierten Probanden vergeben.

Der Prüfarzt führt eine vertrauliche Probandenidentifikationsliste, in der die Screening-Nummer mit dem vollen Probandennamen verbunden ist. Zu dieser Liste hat nur das lokale Studienteam und der klinische Monitor Zugriff.

In einem *Screening/Enrolment Log* werden alle Probanden, die schriftlich der Untersuchungsteilnahme eingewilligt haben, erfasst. In dieser Liste werden Screening- und Random-Nummer, Alter und Geschlecht des Probanden, Datum der Screening-Visite, Datum der Randomisierung und falls erforderlich Gründe für einen Nicht-Einschluss dokumentiert. Ebenfalls werden das Datum der Beendigung der klinisch wissenschaftlichen Untersuchung sowie Informationen zum Beendigungsgrund dokumentiert. Das *Screening/Enrolment Log* und die Probandenidentifikationsliste müssen im ISF abgeheftet werden und für klinische Monitore sowie ggf. Auditoren und Inspektoren zugänglich sein.

Die Originalakten können von Monitoren, Auditoren und Inspektoren eingesehen werden.

Alle Information über die klinisch wissenschaftliche Untersuchung sowie die Untersuchungsdaten und -ergebnisse sind von allen beteiligten Personen streng vertraulich zu behandeln.

## Datenerhebung / Dokumentationsbögen

Die Datenerhebung erfolgt anhand von Papier-CRF, die vom Sponsor, bevollmächtigte Vertreterin des Sponsors (gleichzeitig LKP) Prof. Dr. med. M. Worm zur Verfügung gestellt werden. Die CRF werden mit der entsprechenden Screening- oder Random-Nummer versehen.

Das Original ist für die bevollmächtigte Vertreterin des Sponsors, indem Fall wird es zusammen mit der Source Data gelagert.

Alle aufgrund des Protokolls zu erhebenden Daten werden mit Kugelschreiber sorgfältig und kontinuierlich von einer autorisierten Person (definiert im „Personal-Log“) im CRF erfasst. Bleistifteintragungen sind nicht erlaubt. Korrekturen sind wie folgt vorzunehmen: Der falsche Eintrag wird mit einer einfachen Linie durchgestrichen, die korrekte Information daneben eingetragen und vom Prüfarzt bzw. einer autorisierten Person mit Datum paraphiert (Namenskürzel) und ggf. mit Angabe des Grundes der Korrektur versehen. Der ursprüngliche Eintrag muss erkennbar bleiben, d. h. keine Benutzung von Korrekturflüssigkeiten, Klebeetiketten oder ähnlichem.

Wenn eine Untersuchung nicht durchgeführt wurde, wird dies mit “n.d.” (not done - nicht durchgeführt) angegeben.

Wenn eine Angabe nicht verfügbar ist, wird dies mit “n.a.” (*not available* – nicht verfügbar) angegeben.

## Datenverarbeitung

Grundsätzlich werden fehlende oder unplausible Daten im Rahmen des Monitorings mit dem Prüfarzt geklärt und erforderliche Ergänzungen und Korrekturen in den CRF GCP-gerecht vorgenommen (vgl. Kap 13.2).

In der Studienzentrale (hier: gleichzeitig Prüfzentrum) werden die Daten per EDV erfasst. Eine doppelte Dateneingabe ist nicht vorgesehen. Während der Eingabe werden die Daten wiederholt auf ihre Richtigkeit durch 2 unabhängige Personen überprüft. Nicht plausible oder fehlende Daten können nach Rücksprache mit dem Prüfarzt korrigiert bzw. ergänzt werden. Die Korrekturbelege werden zusammen mit den CRF oder im ISF aufbewahrt. Die Beendigung der Dateneingabe wird dokumentiert.

Erst nach Abschluss dieser Arbeiten wird die Datenbank geschlossen und im Anschluss daran der biometrischen Auswertung zugeführt. Der Verlauf muss dokumentiert und im *Trial Master File* (TMF) abgeheftet werden.

Für die Auswertungen wird SPSS in aktueller Version verwendet.

# Statistische Analyse

## Fallzahlschätzung

Gesamt: 36 Probanden verteilt auf 4 Arme:

- Verum i.m. (n=12)
- Verum s.c. (n=12)
- Placebo i.m. (n=6)
- Placebo s.c. (n=6)

Hinzu kommen noch 4 Probanden (pro Gruppe n=1), die antizipiert die Studie nicht abschließen werden (drop-out).

Da in der Placebogruppe keine immunologische Wirkung erwartet wird, kann diese für die Analyse zusammengefasst werden. Für die Fallzahlschätzung ergeben sich somit folgende 3 Gruppen:

Gruppe 1: Verum i.m.

Gruppe 2: Verum s.c.

Gruppe 3: Placebo i.m. und Placebo s.c.

Der interressierende Wert je Patient errechnet sich aus:

“Anteil CD38^+^-Zellen je B-Zellen Messung nach 28 Tagen /Visite 8“

- (minus)

“Anteil CD38^+^-Zellen je B-Zellen Baseline /Visite 1“ = Δ (in %)

Für den Gruppenmittelwert aus den Einzelwerten der Probanden wurde für jede Gruppe eine Annahme getroffen [%].

- Gruppe 1: μ _Δ1_= 1,4
- Gruppe 2: μ _Δ2_= 1,4
- Gruppe 3: μ _Δ3_= 0

Hierbei wurden für Gruppe 1 und 2 gleiche Mittelwerte angenommen. Trotzdem werden diese Gruppen nicht zusammengefasst, da dies keine gesicherten Annahme ist und die explorative Auswertung ggf. Unterschiede aufzeigen könnte.

Die Standardabweichung aller 3 Gruppen wurde mit σ_1,2,3_=1 angenommen.

Die Mittelwerte und Standardabweichungen entstammen eigenen bislang unpublizierten Untersuchungen, die sich gegenwärtig im Publikationsprozess befinden.

Die Fallzahl wurde für folgende Gruppenvergleiche errechnet:

Fallzahl Hypothesentest I (einseitig)

Gruppe 1 (Verum i.m.) vs. Gruppe 3 (Placebo i.m. und s.c.)

Mann-Whitney Test (Nonparametric Adjust. Normal Distribution)

H_0_: μ _Δ1_= μ _Δ3_

H_1_: μ _Δ1_>μ _Δ3_

🡪Für Gruppe 1 vs. Gruppe 3 ergibt sich bei angenommener β=0.2 (Power=80%) und α_1_=0,0125 eine Fallzahl von je 12 Probanden:

- 12 Probanden Verum i.m.
- 6 (Placebo i.m.) + 6 (Placebo s.c.) = 12 Probanden

Fallzahl Hypothesentest II (einseitig)

Gruppe 2 (Verum s.c.) vs. Gruppe 3 (Placebo i.m. und s.c.)

Mann-Whitney Test (Nonparametric Adjust. Normal Distribution)

H_0_: μ _Δ2_= μ _Δ3_

H_1_: μ _Δ2_>μ _Δ3_

🡪Für Gruppe 2 vs. Gruppe 3 ergibt sich bei angenommener β=0.2 (Power=80%) und α_2_=0,0125 eine Fallzahl von je 12 Probanden

- 12 Probanden Verum s.c.
- 6 (Placebo i.m.) + 6 (Placebo s.c.) = 12 Probanden

**Da die Größen der Mittelwerte/SD nur geschätzte Annahmen sind, werden die errechneten p-Werte nur explorativ betrachtet. Die Powerberechnung dient demnach nur zur Orientierung bei der Wahl der Fallzahl.**

Um 36 auswertbare Patienten zu erhalten, werden unter Berücksichtigung einer Drop-out-rate von 10% in die Studie 40 Patienten eingeschlossen. Die Anzahl zu screenender Patienten liegt bei 50 Patienten, da angenommen wird, dass 80% der gescreenten Patienten die Einschlusskriterien erfüllen.

## Statistische Auswertung

Safety population: umfasst alle Probanden die randomisiert wurden und die Studienmedikation (Verum oder Placebo) erhalten haben. Probanden, die direkt nach der Randomisierung ihr Einverständnis zurückgezogen haben, gehören nicht in diesen Datensatz.

Efficacy population: enthält alle randomisierten Probanden, die protokollgemäß behandelt wurden. Ausgeschlossen werden Probanden aus der efficacy population, wenn mind. eines der folgenden Kriterien erfüllt ist:

• Ein- oder Ausschlusskriterien nach Randomisierung verletzt

• Incompliance in Bezug auf Vermeidung von UV-Exposition / Vitamin D-Einnahme.

Die Auswertung erfolgt analog zur oben dargestellten Fallzahlschätzung:

**Analysen**

Hypothesentest I.)

Gruppe (Verum i.m./s.c., n=24) vs. Gruppe 3 (Placebo i.m./s.c., n=12)

Mann-Whitney Test (unabh. Gruppen)

H_0_: μ_Verum_= μ_Placebo_ (bzw. es spricht nichts gegen die Gleichheit der Verteilungen)

H_1_: μ_verum_>μ_Placebo_ (bzw. Gleichheit der Verteilungen wird abgelehnt)

🡪 Es wird einseitig zum Niveau α=0,025 getestet

Hypothesentest II.)

Gruppe 1 (Verum i.m., n=12) vs. Gruppe 3 (Placebo i.m. und s.c., n=12)

Mann-Whitney Test (unabh. Gruppen)

H_0_: μ_1_= μ_3_ (bzw. es spricht nichts gegen die Gleichheit der Verteilungen)

H_1_: μ_1_>μ_3_ (bzw. Gleichheit der Verteilungen wird abgelehnt)

🡪 Es wird einseitig zum Niveau α_1_=0,0125 getestet

Hypothesentest III.)

Gruppe 2 (Verum s.c., n=12) vs. Gruppe 3 (Placebo i.m. und s.c., n=12)

Mann-Whitney Test (unabh. Gruppen)

H_0_: μ_2_= μ_3_ (bzw. es spricht nichts gegen die Gleichheit der Verteilungen)

H_1_: μ_2_>μ_3_ (bzw. Gleichheit der unabh. Verteilungen wird abgelehnt)

🡪 Es wird einseitig zum Niveau α_2_=0,0125 getestet

In der Summe wird somit nicht das globale Signifikanzniveu von für einseitige Tests eingehalten. Da es sich um keine konfirmatorische Studie handelt, werden die errechneten p-Werte nur explorativ betrachtet. Weiterhin werden die Werte in den Gruppen mittels Histogrammen und Boxplots dargestellt. In Abhängigkeit der Verteilung werden Mittelwert und Standardabweichung oder Median etc. berechnet, sowie Häufigkeitstabellen erstellt.

Weiterhin wird die geschlechtsspezifische Stratifizierung statistisch berücksichtigt, in dem eine Auswertung mittels nichtparametrischer Kovarianzanalyse durchgeführt wird. Das Geschlecht ist hierbei die Kovariable/Kovariate. Diese Betrachtung der Ergebnisse der ANCOVA erfolgt rein explorativ.

**Weitere Analysen**

Die sekundären Endpunkte werden ebenfalls in Abhängigkeit der Verteilung (Mittelwert und Standardabweichung oder Median, sowie Min-/Maximalwerte und Quartile etc.) zu jedem Untersuchungszeitpunkt ausgewertet. Außerdem werden Häufigkeitstabellen erstellt. Um mögliche Zusammenhänge einzelner Variablen zu untersuchen, wird Spearmans Rangkorrelationskoeffizient genutzt.

- Anteil im Blut zirkulierender CD38^+^ B-Zellen im vorher-nachher und Placebo Vergleich bei Vitamin D3-Gabe i.m., bzw. s.c. zu den übrigen Zeitpunkten [%]
- Anteile von T-Zellen und myeloiden antigenpräsentierenden Zellen (APZs) im Blut, die klassische Vitamin D3-induzierte Oberflächenmarker aufweisen (CD38^+^CD4^+^ T-Zellen, HLA-DR^low^CD14^+^ Monozyten) [%]
- Veränderungen im T-Zell-Phänotyp (Anteil naiver und Gedächtniszellen) sowie Zytokinprofil (Häufigkeit von IFN-γ, IL-17, IL-4 sowie IL-10 produzierender CD4^+^ T Helferzellen im Blut) [%]
- Veränderungen im B-Zell-Phänotyp (Anteil naiver und Gedächtniszellen) [%]
- verändertes Profil der Monozytenaktivierung (Häufigkeit HLA-DR^low^CD14^+^ Monozyten sowie CD16^high^CD14^+^ im Blut) [%]
- Veränderungen in der humoralen Immunantwort (anti-EBV-, anti-CMV-, anti-VZV-Immunglobulin im Serum) [units/L]
- Pharmakokinetik (Vitamin D3 Metabolite im Serum) [ng/ml]
- Sicherheit (Blutwerte: Ca, P, Krea, ALT, GGT, diff-BB.)
- Verträglichkeit (Visuelle Analogskala)

Die Ergebnisse werden genutzt, um Hypothesen für spätere Studien mit konfirmatorischen Fragestellungen zu generieren.

# Berichterstattung

Die Erstellung eines integrierten Abschlussberichtes erfolgt innerhalb eines Jahres nach Beendigung der klinisch wissenschaftlichen Untersuchung (gemäß GCP-V §13 Absatz 9). Eine Synopse wird durch den Sponsor, bevollmächtigte Vertreterin des Sponsors (gleichzeitig LPK) Prof. Dr. med. M. Worm dem BfArM und der Ethik-Kommission des Landes Berlin übermittelt. Der Bericht enthält alle wesentlichen Ergebnisse der klinisch wissenschaftlichen Untersuchung, einschließlich dem statistischen Bericht, Einzelwerttabellen und den Schlussfolgerungen.

# Publikation

Die Veröffentlichung der Studienergebnisse erfolgt unabhängig davon, wie die Ergebnisse ausfallen.

Das Protokoll ist Eigentum der bevollmächtigten Vertreterin des Sponsors (gleichzeitig LKP) Prof. Dr. med. M. Worm. Die beteiligten Personen für die Veröffentlichung der Ergebnisse liegen in ihrer Verantwortung.

# Ethische, rechtliche und verwaltungstechnische Aspekte

## Rechtliche Voraussetzungen für die Studie

**Votum der Ethikkommission (gem. AMG § 42 (1) und GCP-V § 7)**

Protokoll, Probandeninformation und Einwilligungserklärung werden der Ethik-Kommission des Landes Berlin zur Begutachtung vorgelegt. Die klinische Untersuchung wird erst nach Erhalt des zustimmenden Votums begonnen.

Die Ethik-Kommission des Landes Berlin wird vom Sponsor, der bevollmächtigten Vertreterin des Sponsors (gleichzeitig LKP) Prof. Dr. med. M. Worm über alle Änderungen im Protokoll (gem. GCP-V § 10) und über alle Ereignisse, die die Sicherheit der Probanden beeinträchtigen könnten, umgehend informiert. Ferner wird die Ethik-Kommission des Landes Berlin über alle der bevollmächtigten Vertreterin des Sponsors bekannt gewordenen SUSARs sowie über das reguläre oder vorzeitige Ende der klinischen Untersuchung unterrichtet.

Alle im Prüfzentrum beteiligten Prüfärzte sind verpflichtet, sich ebenfalls bei der Ethik-Kommission des Landes Berlin anzumelden (Einreichen der Qualifikationsnachweise), bevor sie Probanden in die klinische Untersuchung aufnehmen.

**Genehmigung der Bundesoberbehörde (gem. AMG § 42 (2) und GCP-V § 7)**

Die klinische Untersuchung wird dem Bundesinstitut für Arzneimittel und Medizinprodukte (BfArM) zur Genehmigung vorgelegt. Mit der klinisch wissenschaftlichen Untersuchung wird erst dann begonnen, wenn diese Genehmigung vorliegt.

**Meldung bei den Landesbehörden (gem. AMG § 67)**

Die Durchführung dieser klinisch wissenschaftlichen Untersuchung wird den zuständigen Behörden (für Berlin: LaGeSo) gemeldet. Der Sponsor, bevollmächtigte Vertreterin des Sponsors (gleichzeitig LKP) Prof. Dr. med. M. Worm und sämtliche Prüfärzte sind dort namentlich zu nennen.

**Probandeninformation und Einwilligungserklärung**

Aufklärung der Teilnehmer

Vor Beginn jeglicher untersuchungsspezifischer Maßnahmen wird jeder Teilnehmer vom behandelnden Prüfarzt über Wesen, Ziele, erwartete Vorteile und mögliche Risiken der klinisch wissenschaftlichen Untersuchung mündlich und schriftlich aufgeklärt.

Einwilligung zur Teilnahme an der klinisch wissenschaftlichen Untersuchung

Jeder Proband muss seine schriftliche Einwilligung zur Teilnahme an der klinisch wissenschaftlichen Untersuchung erklären. Dem Proband muss dabei ausreichend Zeit und Gelegenheit gegeben werden, um vor der Einleitung von untersuchungsspezifischen Maßnahmen über seine Teilnahme zu entscheiden und offene Fragen zu klären.

Die Einwilligungserklärung wird vom Teilnehmer und vom behandelnden Prüfarzt unterzeichnet und eigenhändig datiert. Ist der Teilnehmer einwilligungsfähig aber nicht in der Lage, eigenhändig zu unterschreiben, muss ein Zeuge die erfolgte mündliche Aufklärung durch Unterschrift bestätigen.

Die Einwilligungserklärung enthält den Satz: “Ich erkläre hierdurch, dass ich bereit bin, an der klinisch wissenschaftlichen Untersuchung teilzunehmen.“, welcher die eigenständige Entscheidung der Teilnahme des Probanden widerspiegelt. Durch den Satz: „Ich erkläre hierdurch, dass mir bekannt ist, dass meine Teilnahme an der klinisch wissenschaftlichen Untersuchung völlig freiwillig ist und dass ich diese Einwilligung jederzeit und ohne Angabe von Gründen widerrufen kann, ohne dass mir daraus Nachteile für meine weitere Behandlung entstehen.“, ist dem Probanden klar, dass seine Einwilligung freiwillig ist und er diese jeder Zeit zurückziehen kann.

Zudem stimmt der Teilnehmer mit seiner Einwilligung zu, dass er damit einverstanden ist, dass seine personenbezogenen Daten in pseudonymisierter Form an Dritte, u.a. an die zuständige Ethik-Kommission des Landes Berlin und das BfArM weiter gegeben werden.

Der Proband erhält eine Kopie der eigenständig und vom Prüfarzt unterschrieben und datierten Einwilligungserklärung. Das Original verbleibt im Prüfzentrum und wird im ISF abgeheftet.

Die Probandeninformation und die Einwilligungserklärung liegen als separate Dokumente vor und werden der Ethik-Kommission des Landes Berlin zur zustimmenden Bewertung vorgelegt. Beide Dokumente wurden gemäß dem AMG, der GCP‑V und den geltenden gesetzlichen Bestimmungen sowie nach den Empfehlungen der Deklaration von Helsinki und den ICH-GCP-Richtlinien erstellt.

**Probandenversicherung**

Für die vorliegende klinisch wissenschaftliche Untersuchung wird eine Versicherung (gem. AMG § 40 Absatz 1 Satz 3 Nr. 8) abgeschlossen.

**Datenschutz**

Die Probanden werden darüber informiert, dass ihre personenbezogenen Daten in pseudonymisierter Form gespeichert und für wissenschaftliche Auswertungen (z.B. Publikationen) verwendet werden. Die Probanden haben das Recht, über die gespeicherten Daten informiert zu werden. Sie werden auch darüber aufgeklärt, dass ihre pseudonymisierten Daten im Rahmen der gesetzlichen Meldepflichten zur Arzneimittelsicherheit an die zuständige Ethik-Kommission des Landes Berlin und dem BfArM weitergegeben werden und zum Zweck der Qualitätssicherung alle Daten auch vom Monitor und Auditor eingesehen werden dürfen. Probanden, die dieser Weitergabe nicht zustimmen dürfen an der klinisch wissenschaftlichen Untersuchung nicht teilnehmen.

## Aufbewahrung der Daten und Zugang zu den Daten

Die Originale aller zentralen Studiendokumente und aller angefallenen administrativen Dokumente (Schriftverkehr mit Ethik-Kommission des Landes Berlin, BfArM und LaGeSo) werden in der Studienzentrale, hier: Prüfzentrum für mindestens 10 Jahre nach Abschluss der klinischen Untersuchung (GCP-V § 13(10)) aufbewahrt.

Da hier Studienzentral = Prüfzentrum werden auch die Probandenidentifikationsliste, die unterschriebenen Einwilligungserklärungen, die Original-CRFs, die Originaldaten der Prüfungsteilnehmer (Source Data), etc. für die oben genannte Zeit aufbewahrt.

# Literaturverzeichnis

Das vorliegende Protokoll wurde mit der Hilfe eines Masterprotokolls, herausgegeben vom Koordinationszentrum für Klinische Studien (KKS) Charité für Therapiestudien nach dem Arzneimittelgesetz (AMG) verfasst.

1. Liu PT, Stenger S, Li H, et al. Toll-like receptor triggering of a vitamin D-mediated human antimicrobial response. Science. 2006;311:1770-1773.

2. Holick MF. Vitamin D deficiency. N Engl J Med. 2007;357:266-281.

3. Hintzpeter B, Mensink GB, Thierfelder W, Muller MJ, Scheidt-Nave C. Vitamin D status and health correlates among German adults. Eur J Clin Nutr. 2008;62:1079-1089.

4. Vieth R. What is the optimal vitamin D status for health? Prog Biophys Mol Biol. 2006;92:26-32.

5. Kreutz M, Andreesen R, Krause SW, Szabo A, Ritz E, Reichel H. 1,25-dihydroxyvitamin D3 production and vitamin D3 receptor expression are developmentally regulated during differentiation of human monocytes into macrophages. Blood. 1993;82:1300-1307.

6. Penna G, Amuchastegui S, Giarratana N, et al. 1,25-Dihydroxyvitamin D3 selectively modulates tolerogenic properties in myeloid but not plasmacytoid dendritic cells. J Immunol. 2007;178:145-153.

7. Sigmundsdottir H, Pan J, Debes GF, et al. DCs metabolize sunlight-induced vitamin D3 to 'program' T cell attraction to the epidermal chemokine CCL27. Nat Immunol. 2007;8:285-293.

8. Heine G, Niesner U, Chang HD, et al. 1,25-dihydroxyvitamin D(3) promotes IL-10 production in human B cells. Eur J Immunol. 2008;38:2210-2218.

9. Chen S, Sims GP, Chen XX, Gu YY, Chen S, Lipsky PE. Modulatory effects of 1,25-dihydroxyvitamin d3 on human B cell differentiation. J Immunol. 2007;179:1634-1647.

10. Dimeloe S, Richards DF, Urry ZL, et al. 1alpha,25-Dihydroxyvitamin D3 promotes CD200 expression by human peripheral and airway-resident T cells. Thorax. 2012.

11. Xystrakis E, Kusumakar S, Boswell S, et al. Reversing the defective induction of IL-10-secreting regulatory T cells in glucocorticoid-resistant asthma patients. J Clin Invest. 2006;116:146-155.

12. Smolders J, Peelen E, Thewissen M, et al. Safety and T cell modulating effects of high dose vitamin D3 supplementation in multiple sclerosis. PLoS One. 2010;5:e15235.

13. Urry Z, Xystrakis E, Richards DF, et al. Ligation of TLR9 induced on human IL-10-secreting Tregs by 1alpha,25-dihydroxyvitamin D3 abrogates regulatory function. J Clin Invest. 2009;119:387-398.

14. Stoeckler JD, Stoeckler HA, Kouttab N, Maizel AL. 1alpha,25-Dihydroxyvitamin D3 modulates CD38 expression on human lymphocytes. J Immunol. 1996;157:4908-4917.

15. Yamanaka K, Dimitroff CJ, Fuhlbrigge RC, et al. Vitamins A and D are potent inhibitors of cutaneous lymphocyte-associated antigen expression. J Allergy Clin Immunol. 2008;121:148-157 e143.

16. Shirakawa AK, Nagakubo D, Hieshima K, Nakayama T, Jin Z, Yoshie O. 1,25-dihydroxyvitamin D3 induces CCR10 expression in terminally differentiating human B cells. J Immunol. 2008;180:2786-2795.

17. Fritsche J, Mondal K, Ehrnsperger A, Andreesen R, Kreutz M. Regulation of 25-hydroxyvitamin D3-1 alpha-hydroxylase and production of 1 alpha,25-dihydroxyvitamin D3 by human dendritic cells. Blood. 2003;102:3314-3316.

18. Mora JR, Iwata M, von Andrian UH. Vitamin effects on the immune system: vitamins A and D take centre stage. Nat Rev Immunol. 2008;8:685-698.

19. Munger KL, Levin LI, Hollis BW, Howard NS, Ascherio A. Serum 25-hydroxyvitamin D levels and risk of multiple sclerosis. Jama. 2006;296:2832-2838.

20. Holick MF. Resurrection of vitamin D deficiency and rickets. J Clin Invest. 2006;116:2062-2072.

21. Peterlik M, Cross HS. Vitamin D and calcium deficits predispose for multiple chronic diseases. Eur J Clin Invest. 2005;35:290-304.

22. Vieth R. Vitamin D supplementation, 25-hydroxyvitamin D concentrations, and safety. Am J Clin Nutr. 1999;69:842-856.

23. Vieth R. Vitamin D toxicity, policy, and science. J Bone Miner Res. 2007;22 Suppl 2:V64-68.

24. Heine G, Lahl A, Muller C, Worm M. Vitamin D deficiency in patients with cutaneous lupus erythematosus is prevalent throughout the year. Br J Dermatol. 2010;163:863-865.

25. Renne J, Werfel T, Wittmann M. High frequency of vitamin D deficiency among patients with cutaneous lupus erythematosus. Br J Dermatol. 2008;159:485-486.

26. Brehm JM, Celedon JC, Soto-Quiros ME, et al. Serum vitamin D levels and markers of severity of childhood asthma in Costa Rica. Am J Respir Crit Care Med. 2009;179:765-771.

27. Brehm JM, Schuemann B, Fuhlbrigge AL, et al. Serum vitamin D levels and severe asthma exacerbations in the Childhood Asthma Management Program study. J Allergy Clin Immunol. 2010;126:52-58 e55.

28. Sidbury R, Sullivan AF, Thadhani RI, Camargo CA, Jr. Randomized controlled trial of vitamin D supplementation for winter-related atopic dermatitis in Boston: a pilot study. Br J Dermatol. 2008;159:245-247.

29. Peroni DG, Piacentini GL, Cametti E, Chinellato I, Boner AL. Correlation between serum 25-hydroxyvitamin D levels and severity of atopic dermatitis in children. Br J Dermatol. 2011;164:1078-1082.

30. Zittermann A, Dembinski J, Stehle P. Low vitamin D status is associated with low cord blood levels of the immunosuppressive cytokine interleukin-10. Pediatr Allergy Immunol. 2004;15:242-246.

31. Heine G, Anton K, Henz BM, Worm M. 1alpha,25-dihydroxyvitamin D3 inhibits anti-CD40 plus IL-4-mediated IgE production in vitro. Eur J Immunol. 2002;32:3395-3404.

32. Milovanovic M, Heine G, Hallatschek W, Opitz B, Radbruch A, Worm M. Vitamin D receptor binds to the epsilon germline gene promoter and exhibits transrepressive activity. J Allergy Clin Immunol. 2010;126:1016-1023, 1023 e1011-1014.

33. Hartmann B, Heine G, Babina M, et al. Targeting the vitamin D receptor inhibits the B cell-dependent allergic immune response. Allergy. 2011;66:540-548.

34. Wittke A, Weaver V, Mahon BD, August A, Cantorna MT. Vitamin D receptor-deficient mice fail to develop experimental allergic asthma. J Immunol. 2004;173:3432-3436.

35. May E, Asadullah K, Zugel U. Immunoregulation through 1,25-dihydroxyvitamin D3 and its analogs. Curr Drug Targets Inflamm Allergy. 2004;3:377-393.

36. Kimball SM, Ursell MR, O'Connor P, Vieth R. Safety of vitamin D3 in adults with multiple sclerosis. Am J Clin Nutr. 2007;86:645-651.

37. Stubbs JR, Idiculla A, Slusser J, Menard R, Quarles LD. Cholecalciferol supplementation alters calcitriol-responsive monocyte proteins and decreases inflammatory cytokines in ESRD. J Am Soc Nephrol. 2010;21:353-361.

38. Heine G, Drozdenko. G, Lahl A, et al. Efficient tetanus toxoid immunization on vitamin D supplementation. Eur J Clin Nutr. 2011;65:329-334.

39. Mahon BD, Gordon SA, Cruz J, Cosman F, Cantorna MT. Cytokine profile in patients with multiple sclerosis following vitamin D supplementation. J Neuroimmunol. 2003;134:128-132.

40. Schleithoff SS, Zittermann A, Tenderich G, Berthold HK, Stehle P, Koerfer R. Vitamin D supplementation improves cytokine profiles in patients with congestive heart failure: a double-blind, randomized, placebo-controlled trial. Am J Clin Nutr. 2006;83:754-759.

41. Heaney RP, Davies KM, Chen TC, Holick MF, Barger-Lux MJ. Human serum 25-hydroxycholecalciferol response to extended oral dosing with cholecalciferol. Am J Clin Nutr. 2003;77:204-210.

# Anhänge

**Anhang 1 Pharmakologisch-toxikologisches Gutachten Fa. HEYL 2008**

Separates Dokument (zur Einreichung bei beim BfArM)

**Anhang 2 Klinische Gutachten Fa. HEYL 2008**

Separates Dokument (zur Einreichung bei beim BfArM)

**Anhang 3 Etiketten der Studienmedikation**

| ZUR KLINISCHEN PRÜFUNG BESTIMMT  Sponsor: Charité – Universitätsmedizin Berlin, Charitéplatz 1, 10117 Berlin  LKP und bevollmächtigte Vertreterin des Sponsors: Prof. Dr. M. Worm Tel. 030 450 518105  EudraCT-Nr. 2012-003217-33 Prüfplancode: ViDImmun  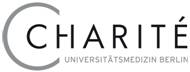 Apotheke I Augustenburger Platz 1 I 13353 Berlin I 030-450 56 11 04 |
| --- |

**Anhang 4 Anzeigetext zur Unterstützung der Probandenrekrutierung**


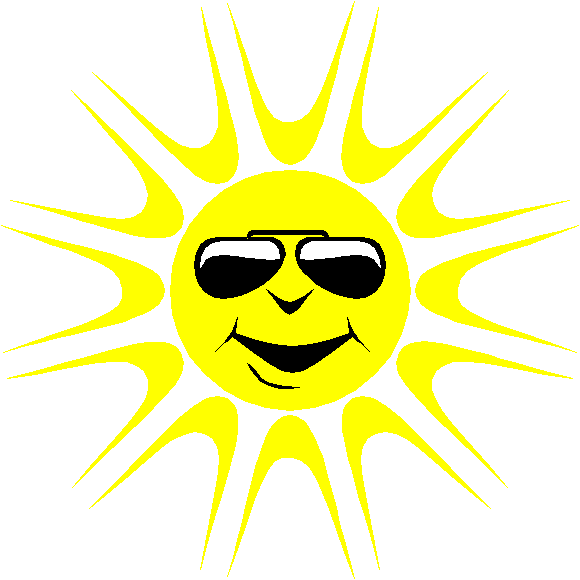


SONNENSCHEIN - HORMON

Das Allergie-Centrum-Charité sucht
männliche und weibliche Teilnehmer im Alter zwischen 18 und 60 Jahren für eine
**wissenschaftliche Untersuchung zur immunologischen Wirkung von Vitamin D**.

Es wird eine Hochdosis-Vitamin D Präparation gegeben im Abstand und darunter im Blut die Immunantwort gemessen.

Sie werden gründlich medizinisch aufgeklärt und durchgehend ärztlich betreut.
Eine Aufwandsentschädigung wird Ihnen gewährt.

Sollten Sie Interesse an einer Teilnahme haben, nehmen Sie

Kontakt mit uns auf. Wir freuen uns auf Ihren Anruf!

Allergie-Centrum-Charité

Studien-Telefon: 030-450 518 003 u. -417 (Mo-Do 12-14 Uhr, Frau Lungwitz)

Studien-E-Mail: acc-studien@charite.de

**Anhang 5 Referenzbereiche / Normwerte**

(Charité - Universitätsmedizin Berlin, Institut für Laboratoriumsmedizin, Klinische Chemie und Pathobiochemie, seit 01.01.2011 Labor Berlin – Charité Vivantes GmbH)

**Hämatologie:**

Leukozyten (EDTA-Blut) 4,50 - 11,00/nl

Hämoglobin (EDTA-Blut) 12,0 - 15,7 g/dl (Frauen) und 14,0 - 17,5 g/dl (Männer)

Thrombozyten (EDTA-Blut) 150 - 400/nl

Erythrozyten-(EDTA-Blut) 3,90 - 5,40/pl (Frauen) und 4,60 - 6,20/pl (Männer)

**Klinische Chemie:**

Kalzium (Heparin-Blut) 2,15 - 2,65 mmol/l

Phosphat (Heparin-Blut) 0,80 - 1,50 mmol/l

Kreatinin (Heparin-Blut) <1,00 mg/dl (Frauen) und <1,20 mg/dl (Männer)

ALT (Heparin-Blut) < 34 U/l (Frauen) und < 45 U/L (Männer)

GGT (Heparin-Blut) < 38 U/l (Frauen) und < 55 U/l (Männer)

25-Hydroxyvitamin D (Serum) 50,0 - 250,0 nmol/l

**Vital-Parameter:**

Puls: Sitzend: 60 - 80 Schläge/min

Blutdruck: systolisch 100 - 135 mmHg

diastolisch 60 - 85 mmHg

**Anhang 6 Verantwortlichkeiten**

| **Sponsor** | Charité – Universitätsmedizin Berlin |
| --- | --- |
| **Bevollmächtigte Vertreterin des Sponsors und Leiterin der klinischen Prüfung** | Prof. Dr. med. Margitta Worm  Charité – Universitätsmedizin Berlin  Klinik für Dermatologie, Venerologie und Allergologie  Allergie-Centrum-Charité (ACC)  Charitéplatz 1, 10117 Berlin  Tel.: +49 (0)30-450 518 105  Fax: +49 (0)30-450 518 931  [margitta.worm@charite.de](mailto:margitta.worm@charite.de) |
| **Betreuende Prüfärzte** | Dr. med. Guido Heine  Charité – Universitätsmedizin Berlin  Klinik für Dermatologie, Venerologie und Allergologie  Allergie-Centrum-Charité (ACC)  Charitéplatz 1, 10117 Berlin  Tel.: +49 (0)30-450 618 305  Fax: +49 (0)30-450 518 968  [guido.heine@charite.de](mailto:guido.heine@charite.de)  Dr. Dirk Tomsitz  Charité – Universitätsmedizin Berlin  Klinik für Dermatologie, Venerologie und Allergologie  Allergie-Centrum-Charité (ACC)  Charitéplatz 1, 10117 Berlin  Tel.: +49 (0)30-450 518 759  Fax: +49 (0)30-450 518 968  [dirk.tomsitz@charite.de](mailto:dirk.tomsitz@charite.de)  Katharina Sophie Wylon  Charité – Universitätsmedizin Berlin  Klinik für Dermatologie, Venerologie und Allergologie  Allergie-Centrum-Charité (ACC)  Charitéplatz 1, 10117 003  Fax: +49 (0)30-450 518 968 |
| **Studienstatistiker** | Statistische Planung:  Gerald Splettstößer  Alexander Krannich  Koordinierungszentrum für klinische Studien  Charité – Universitätsmedizin Berlin  Campus Virchow-Klinikum (CVK)  Augustenburger Platz 1  13353 Berlin |
| **Weitere beteiligte Personen und Institutionen, Prüflabore etc.** | Studienkoordination:  Dr. rer. medic. Sabine Dölle  Charité – Universitätsmedizin Berlin  Klinik für Dermatologie, Venerologie und Allergologie  Allergie-Centrum-Charité (ACC)  Charitéplatz 1, 10117 Berlin  Tel.: +49 (0)30-450 518 367  Fax: +49 (0)30-450 518 968  [sabine.doelle@charite.de](mailto:sabine.doelle@charite.de)  Labore der Charité - Universitätsmedizin Berlin:  Institut für Laboratoriumsmedizin, Klinische Chemie und Pathobiochemie, seit 01.01.2011 Labor Berlin – Charité Vivantes GmbH  Prof. Dr. med. Rudolf Tauber  Augustenburger Platz 1, 13353 Berlin  Forschungslabor der Klinik für Dermatologie,  Venerologie und Allergologie  Prof. Dr. med. M. Worm  Charitéplatz 1, 10117 Berlin |
| **Projektpartner** | HEYL Chem.-pharm. Fabrik GmbH & Co. KG  Dr. Johann Ruprecht  Gorzallee 253, 14167 Berlin  Tel.: +49 (0)30-81696-26  Fax: +49 (0)30-8174049  [johann.ruprecht@heyl-berlin.de](mailto:johann.ruprecht@heyl-berlin.de) |
| **Randomisierung, Etikettierung** | Charité - Universitätsmedizin Berlin  Apotheke, Klinische Pharmazie (CVK)  Cornelia Eberhard  Augustenburger Platz 1, 13353 Berlin  Tel.: +49 (0)30-450 561 104  Fax: +49 (0)30-450 561 905  [cornelia.eberhardt@charite.de](mailto:cornelia.eberhardt@charite.de) |

**Anhang 7 sonstiges**

Aus Vieth, R. 1999. Vitamin D supplementation, 25-hydroxyvitamin D concentrations, and safety. Am J Clin Nutr 69:842-856. [^22^](#_ENREF_23)

1. gemäß Vieth R. 2006 Prog Biophys Mol Biol. [↑](#footnote-ref-2)
2. gemäß Vieth R. 2006 Prog Biophys Mol Biol. [↑](#footnote-ref-3)
3. RICHTLINIE 2001/20/EG: (14) Nichtkommerzielle klinische Prüfungen, die von Wissenschaftlern ohne Beteiligung der pharmazeutischen Industrie durchgeführt werden, können einen hohen Nutzen für die betroffenen Patienten haben. Daher sollte die Richtlinie die besondere Situation der Prüfungen berücksichtigen, deren Konzept keine besondere Herstellung oder Verpackung erfordert, falls diese Prüfungen mit Arzneimitteln, für die im Sinne der Richtlinie 65/65/EWG eine Genehmigung für das Inverkehrbringen erteilt wurde und die gemäß den Vorschriften der Richtlinien 75/319/EWG und 91/356/EWG hergestellt oder importiert wurden, durchgeführt werden, und zwar bei Patienten mit denselben Merkmalen wie die, die von dem in der Genehmigung für das Inverkehrbringen genannten Anwendungsgebiet abgedeckt sind. Die Kennzeichnung der Prüfpräparate für derartige Prüfungen sollte den vereinfachten Bestimmungen unterliegen, die in den Leitlinien über gute Herstellungspraxis bei Prüfpräparaten und in der Richtlinie 91/356/EWG niedergelegt sind. [↑](#footnote-ref-4)
4. <http://ec.europa.eu/health/documents/eudralex/vol-4/index_en.htm> [↑](#footnote-ref-5)
